# Exploring the Crystal Landscape of Mandelamide and Chiral Resolution via Cocrystallization

**DOI:** 10.1021/acs.cgd.3c01513

**Published:** 2024-12-10

**Authors:** Shan Huang, Deirbhile Fitzgerald, Samuel A. Koledoye, Stuart G. Collins, Anita R. Maguire, Simon E. Lawrence

**Affiliations:** † School of Chemistry, Analytical and Biological Chemistry Research Facility, SSPC, the SFI Research Centre for Pharmaceuticals, University College Cork, Cork T12 K8AF, Ireland; ‡ School of Chemistry and School of Pharmacy, Analytical and Biological Chemistry Research Facility, SSPC, the SFI Research Centre for Pharmaceuticals, University College Cork, Cork T12 K8AF, Ireland

## Abstract

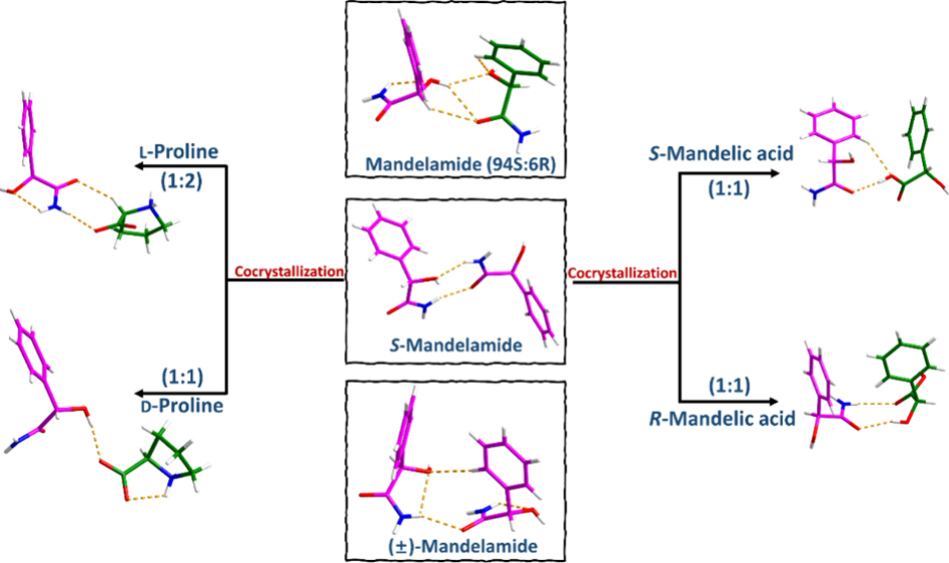

The crystal structures of (±)-mandelamide, *S*-mandelamide, and enantioenriched mandelamide (94 *S* : 6 *R*) were determined. Diastereomeric cocrystal pairs of *S*-mandelamide with both enantiomers of mandelic acid and proline were synthesized. The diastereomeric cocrystal pairs of *S*-mandelamide with *S*/*R*-mandelic acid form 1:1 cocrystals in each case, while the diastereomeric cocrystal pairs of *S*-mandelamide with proline have different stoichiometries. Preliminary investigation of this diastereomeric cocrystal system for chiral resolution shows promise.

## Abstract 2

Diastereomeric cocrystal pairs of *S*-mandelamide with both enantiomers of mandelic acid and proline were synthesized. The proof of concept of chiral resolution was demonstrated for the mandelamide and proline system.

## Introduction

Chirality, a distinct characteristic of objects that cannot be perfectly aligned with their mirror image, is present in various aspects of nature. For example, the standard form of the DNA double helix always twists in a right-handed manner, while snails exhibit left–right asymmetry both internally and externally.^1,2^ A large number of naturally occurring molecules, such as proteins, enzymes, amino acids, carbohydrates, etc., are chiral and contain at least one stereogenic center in the structure, typically tetrahedral (sp^3^-hybridized) carbons with four different substituents,^3^ and the two nonsuperimposable mirror-image forms of chiral molecules are called enantiomers.^4^ A review from 2003 states that approximately 50% of the pharmaceuticals marketed and used in medical treatment are chiral compounds, and 88% among them are administered as racemates.^5^ Different enantiomers of a chiral compound generally possess identical physical and chemical properties in an achiral environment, but they may exhibit significant variations in biological activities. For example, the (*S*,*S*)-(+)-enantiomer of ethambutol is utilized for treating tuberculosis, while the (*R*,*R*)-(−)-enantiomermay lead to blindness.^6^ Nowadays, regulatory authorities require independent pharmacological tests for each enantiomer as well as their combined effects, and only the therapeutically active isomer can be used in a marketed drug product,^7^ consequently, stereochemistry and chiral resolution are of paramount importance in the pharmaceutical industry. The 2001 Nobel Prize in Chemistry was awarded to three scientists for their work in the development of asymmetric synthesis using chiral catalysts in the production of single enantiomer drugs or chemicals.^8^ In spite of the rapid development of asymmetric synthesis in recent years, there are still numerous chiral compounds synthesized as racemates, and then separated by a suitable physical separation approach.^9^ In industry, two main categories of techniques are often applied for chiral resolution. Diastereomeric salt formation and enzymatic or kinetic resolution are two classical technologies, and the modern approach is the use of preparative high-performance liquid chromatography.^10−12^ The main restrictions of the above methods is that sometimes they are impractical and uneconomical.

**Figure 1:**
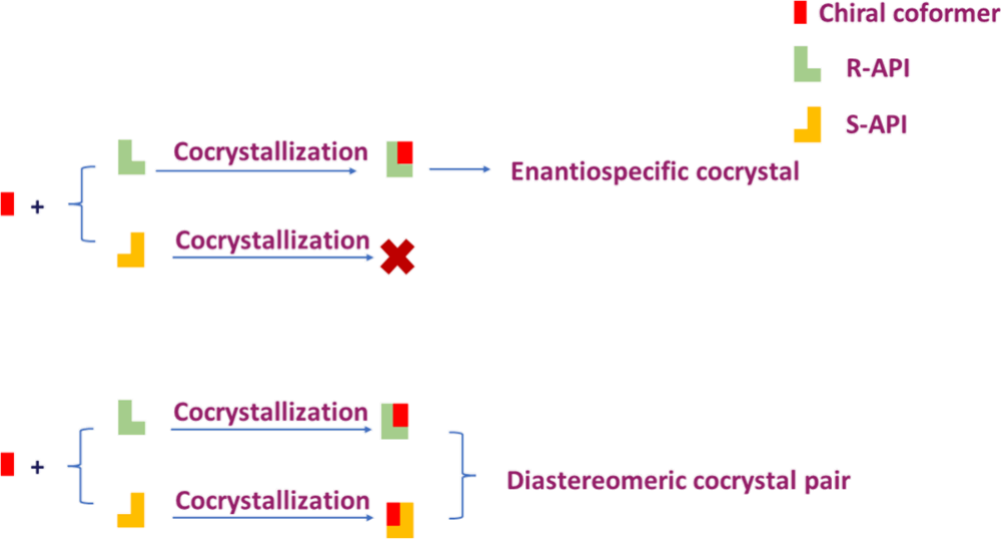
Two possible scenarios of achieving chiral resolution by cocrystal formation (adapted from ref (14) with permission; Copyright 2012 American Chemical Society).

Cocrystallization, the process of producing cocrystals, i.e., crystals with two or more molecular species in a specific stoichiometric ratio within a crystal lattice, has gained increasing attention recently as a feasible strategy to achieve chiral separation.^13−15^ This process enables the formation of new crystalline materials involving two chiral molecules, leading to changes in its physical and physicochemical properties.^16^ This approach involves two possible scenarios when both cocrystallizing components are chiral: (i) the chiral coformer only forms an enantiospecific crystal with one enantiomer of the target compound or (ii) the chiral coformer can form a diastereomeric cocrystal pair with each enantiomer of the target compound. Structural modifications in the supramolecular assembly in enantiospecific cocrystals or diastereomeric cocrystal pairs lead to changes in the crystal lattice energy and related physical and physicochemical properties, enabling separation (Figure 1). Therefore, both possible outcomes can be used to develop a chiral resolution process.

The application of achieving chiral resolution through enantiospecific cocrystal formation in solution was first introduced by Leyssens’s group in 2012,^14^ and developed to include a dual-drug chiral resolution process^17^ and the use of ionic cocrystals.^16,18^ They initially demonstrated that only the *S*-enantiomer of 2-(2-oxopyrrolidin-1-yl) butanamide, which exhibits nootropic activity and is marketed under the name levetiracetam, can cocrystallize with *S*-mandelic acid, while the *R*-enantiomer cannot form a cocrystal with *S*-mandelic acid, leading to 70% of the *S*-enantiomer separated from the racemic mixture in a single cocrystallization step.

Diastereomeric cocrystal systems have been less extensively studied in comparison to enantiospecific systems. Höpfl and colleagues reported a diastereomeric cocrystal pair of *R*/*S*-praziquantel with l-malic acid, and the chiral separation was enabled by phase-decomposition of the *R*-praziquantel-l-malic acid cocrystal due to the different aqueous solubilities of the diastereomeric cocrystals.^19^l-Proline was proven to form diastereomeric cocrystals with both *R*- and *S*-enantiomers of mandelic acid in different stoichiometric ratios, hence, the chiral separation can be attained by simply altering the stoichiometry of the two constituents.^20^

**Figure 2:**
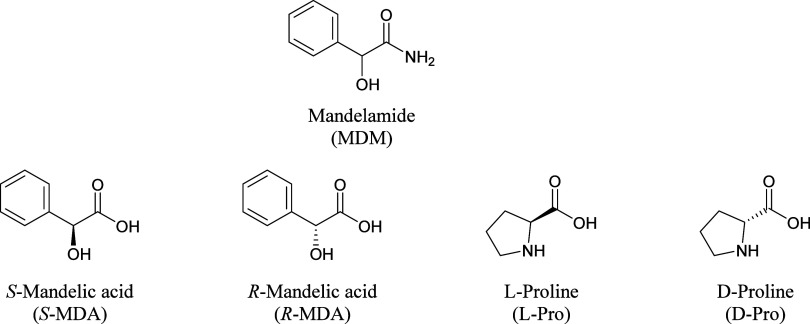
Molecular structures of MDM and coformers present in this work.

Mandelic acid is a widely used compound for forming enantiospecific or diastereomeric cocrystals. The literature and Cambridge Structural Database (CSD) search indicate that approximately 40 cocrystals/salts incorporating mandelic acid with another chiral compound have been documented (Table S11). Somewhat surprisingly, no cocrystals involving mandelamide, the amide derivative of mandelic acid, have been reported or deposited in the CSD,^21^ even though it is an important drug precursor.^22^ In this work, the crystal structure of racemic [(±)**-**MDM], enantiopure mandelamide (*S*-MDM) and enantioenriched MDM (94 *S* : 6 *R*) were identified, and the potential of *S*-MDM as a chiral resolution agent via cocrystallization was considered. Two diastereomeric cocrystal pairs of *S*-MDM with both *R*- and *S*-enantiomers of mandelic acid (MDA) and proline (Pro) (Figure 2) were obtained by both liquid-assisted grinding and slow evaporation, and fully characterized by thermal analysis, X-ray techniques, and FT-IR spectroscopy. To further investigate the diastereomeric behavior of *S*-MDM with the chiral coformers, detailed analyses of crystal structures, motifs and Hirshfeld surfaces were performed.

## Experimental Section

### Materials

*S*-MDA, *R*-MDA, and l-Pro was obtained from Fluorochem and d-Pro from TCI chemicals. (±)**-**MDM was synthesized from (±)**-**MDA using a literature procedure^23^ and was recrystallized from hot ethanol to yield white plates. *S*-MDM was synthesized from *S*-MDA using a similar procedure to that used for (±)-MDM, see the SI. (±)**-**MDA and commercial *S*-MDM were obtained from Sigma-Aldrich. Solvents were purchased from commercial sources and all materials were used as received.

### Liquid-Assisted Grinding (LAG)

LAG experiments were performed by placing a physical mixture of *S*-MDM with each coformer in a 5 mL stainless steel grinding jar along with a 2.5 mm stainless steel grinding ball. After the addition of 30 μL of ethyl acetate the mixture was ground using a Retsch MM400 Mixer mill at a rate of 30 Hz for 30 min. The products obtained were analyzed by powder X-ray diffraction (PXRD). A 1:1 molar ratio of *S*-MDM: coformer was used in all cases. After single crystal analysis, a 1:2 molar ratio of *S*-MDM with l-Pro was used.

#### Crystallization of (±)-MDM

20.5 mg of synthesized (±)-MDM was dissolved in 10 mL of THF by heating. Colorless plate-like crystals were obtained by slowly evaporating the filtered solution at room temperature for 3–5 d.

#### Crystallization of *S*-MDM

20.2 mg of synthesized *S*-MDM was dissolved in 5 mL of MeOH by heating. Colorless plate-like crystals were obtained by slowly evaporating the filtered solution at room temperature for 3–5 d. The bulk commercial sample is identical by PXRD.

#### Crystallization of MDM (94 *S* : 6 *R*)

20.4 mg of the commercial *S*-MDM was dissolved in 10 mL of the solvent mixture THF and toluene (1:1, v/v) by heating. Colorless plate-like crystals of MDM were obtained by slowly evaporating the filtered solution at room temperature for 3–5 d and one crystal was identified by single crystal diffraction as containing 94% *S*-MDM and 6% *R*-MDM. Bulk quantities of MDM (94 *S* : 6 *R*) were obtained by dissolving 100 mg of the commercial *S*-MDM in EtOH at room temperature, and removing the solvent quickly using a rotary evaporator (Büchi, Germany) under a vacuum achieved by a diaphragm pump (Vacuubrand, Germany), with the rotary flask rotating at a speed of 40 rpm while being immersed in a water bath at 50 °C.^24^ The resulting white powdered product was isolated and allowed to dry in the fume hood overnight.

### Crystallization of Cocrystals

The products from the LAG experiments were dissolved in 10 mL of solvent and the filtrate allowed to crystallize by slow evaporation.

#### *S*-MDM-*S*-MDA

22.7 mg of powdered *S*-MDM-*S*-MDA was used in MeOH. Colorless plate-like crystals were harvested after 3–5 d.

#### *S*-MDM-*R*-MDA

20.8 mg of powdered *S*-MDM-*R*-MDA was used in a solvent mixture of MeOH and Et_2_O (1:1, v/v). Colorless needle-like crystals were after 5–7 d.

#### *S*-MDM-l-Pro

31.4 mg of S-MDM-l-Pro was used with the solvent mixture of EtOH and CH_2_Cl_2_ (1:1, v/v). Colorless needle-like crystals were obtained after 3–5 d.

#### *S*-MDM-d-Pro

19.8 mg of S-MDM-d-Pro in the mixed solvent MeOH and THF (1:1, v/v). Colorless needle-like crystals were obtained after 3–5 d.

### Physical Measurements

Powder X-ray Diffraction (PXRD): The PXRD patterns were collected on a STOE STADI MP diffractometer with a Cu Kα radiation (1.540 Å) using a linear position-sensitive detector. The tube voltage and amperage were set at 40 kV and 40 mA respectively. Each sample was scanned between 3.5 and 45.5° 2θ with an increment of 0.05° at a rate of 2° min^–1^. The samples were prepared as transmission foils and the data were viewed via STOE WinXPOW POWDAT software.^25^

Differential Scanning Calorimetry (DSC): DSC was conducted on a TA Instruments Q1000. Samples (1–5 mg) were placed in nonhermetic aluminum pans and scanned in the range of 25 to 200 °C at a heating rate of 10 °C min^–1^ under a continuously purged dry nitrogen atmosphere (flow rate 80 mL min^–1^). The data were viewed and analyzed by TA Universal Analysis software.

FT-IR Spectroscopy (IR): FT-IR spectra were recorded on a PerkinElmer UATR Two spectrophotometer using a diamond attenuated total reflectance accessory over a range of 400–4000 cm^–1^. Four scans were taken at 4 cm^–1^ resolution for each sample, and the spectra were measured over the range of 400–4000 cm^–1^.

Single crystal X-ray diffraction (SCXRD): An optical microscope (Zeiss Stemi 2000) was used to choose a suitable crystal for diffraction. SCXRD data was performed using a Bruker APEX II DUO with monochromated Cu Kα radiation (λ = 1.54178 Å). The structure was solved and refined by the SHELX suite of programs in the Bruker APEX software.^26,27^ All non-hydrogen atoms were refined by using anisotropic displacement parameters while hydrogen atoms were fixed in geometrically calculated positions using the riding model, with C–H = 0.93–0.98 Å, O–H = 0.82 Å and N–H = 0.86–0.89 Å, and Uiso (H) (in the range 1.2–1.5 times Ueq of the parent atom). For MDM (94 *S* : 6 *R*), there is disorder in two of the four crystallographically independent MDM molecules due to the *R*-MDM impurity, which was modeled in two conformations in 88:12 ratio. For *S*-MDM-l-Pro and *S*-MDM-d-Pro cocrystals, there was disorder in the proline carbon that is beta to both the nitrogen and the carbon bonded to the carboxylic acid, which was modeled in two conformations in 50:50 and 85:15 ratios, respectively. PLATON was used for the analysis of potential hydrogen bonds and short ring interactions.^28,29^ Mercury 2022.2.0 and DIAMOND 4.6 were used for viewing structures and creating diagrams.^30^ Crystallographic parameters are listed in Table 1.

**Table 1:** Crystallographic Data for (±)-MDM, *S*-MDM, MDM (94 *S* : 6 *R*), *S*-MDM-*S*-MDA, *S*-MDM-*R*-MDA, *S*-MDM-l-Pro, and *S*-MDM-d-Pro

	(±)-MDM	*S*-MDM	MDM (94 *S*: 6 *R*)	*S*-MDM-*S*-MDA 1:1	*S*-MDM-*R*-MDA 1:1	*S*-MDM-l-Pro 1:2	*S*-MDM-d-Pro 1:1
chemical formula	C_8_H_9_NO_2_	C_8_H_9_NO_2_	C_8_H_9_NO_2_	C_16_H_17_NO_5_	C_16_H_17_NO_5_	C_18_H_27_N_3_O_6_	C_13_H_18_N_2_O_4_
formula weight	151.16	151.16	151.16	303.30	303.30	381.42	266.29
crystal system	monoclinic	orthorhombic	monoclinic	orthorhombic	orthorhombic	orthorhombic	monoclinic
space group	*P*2_1_*/c*	*P*2_1_2_1_2_1_	*P*2_1_	*P*2_1_2_1_2_1_	*P*2_1_2_1_2_1_	*P*2_1_2_1_2_1_	*P*2_1_
*Z*, *Z’*	4, 1	4, 1	8, 4	4, 1	4, 1	4, 1	4, 2
temperature (K)	296(2)	296(2)	296(2)	296(2)	296(2)	296(2)	296(2)
*a* (Å)	15.906(3)	5.5857(5)	8.2111(5)	6.4068(6)	6.9120(11)	5.6311(4)	5.19880(10)
*b* (Å)	5.5263(12)	8.2971(8)	5.9441(4)	8.0493(7)	7.3147(12)	14.9687(11)	24.6214(4)
*c* (Å)	8.5110(15)	16.8366(16)	31.3477(19)	29.633(3)	29.941(4)	22.9618(16)	10.51280(10)
α (deg)	90	90	90	90	90	90	90
β (deg)	91.386(15)	90	95.190(3)	90	90	90	95.4650(10)
γ (deg)	90	90	90	90	90	90	90
volume (Å^3^)	747.9(3)	780.29(13)	1523.73(17)	1528.2(2)	1513.8(4)	1935.5(2)	1339.54(4)
ρ_calc_ (g cm^–3^)	1.342	1.287	1.318	1.318	1.331	1.309	1.320
radiation type	Cu Kα	Cu Kα	Cu Kα	Cu Kα	Cu Kα	Cu Kα	Cu Kα
μ (mm^–1^)	0.806	0.772	0.791	0.823	0.830	0.822	0.819
Reflns measured	4243	4919	18234	9838	12366	11409	17685
*R*_int_	0.054	0.030	0.029	0.028	0.048	0.030	0.023
Reflns independent	1231	1319	5155	2677	2589	3325	4582
significant [*I* > 2σ(*I*)]	899	1312	4982	2662	2367	2861	4502
parameters refined	101	101	423	202	202	255	358
restraints	0	0	4	6	6	4	15
Δρ_max_, Δρ_min_ (e Å^–3^)	0.371, −0.201	0.138, −0.174	0.102, −0.127	0.344, −0.410	0.323, −0.174	0.353, −0.176	0.249, −0.189
*F*(000)	320	320	640	640	640	816	568
*R*_1_ [*I* > 2σ(*I*)]	0.0718	0.0342	0.0277	0.0379	0.0375	0.0383	0.0314
*wR*_2_ (all data)	0.2077	0.0906	0.0727	0.1071	0.0986	0.1113	0.0855
Flack		0.08(5)	0.05(5)	0.03(5)	0.08(10)	0.11(8)	0.10(4)
CCDC	2269509	2269510	2269507	2269512	2269508	2269506	2269511

### Computational Studies

Hirshfeld surface analyses and two-dimensional (2D) fingerprint plots were carried out using the CrystalExplorer 21.5 program.^31^

### Analysis of the Cambridge Structural Database

Searches of the CSD were conducted using ConQuest version 2022.2.0.^32^ The following restrictions were applied: 3D coordinates; single crystal structures only; and organics only.

### Nuclear Magnetic Resonance (^1^H NMR) Analysis

NMR spectra were recorded on either a Bruker Avance 300 MHz NMR spectrometer ^1^H (300 MHz) or on a Bruker Avance 400 MHz NMR spectrometer ^1^H (400 MHz) and ^13^C (100.6 MHz). All spectra were recorded at room temperature (20 °C) in deuterated methanol (*d*_4_-CD_3_OD), using tetramethylsilane (TMS) as an internal standard. Chemical shifts are reported in parts per million (ppm) relative to TMS, and coupling constants are expressed in Hertz (Hz).

### Chiral High-Performance Liquid Chromatography Analysis

The enantiopurity of the commercial *S-*MDM from Sigma-Aldrich, synthesized *S-*MDM and the single crystal of MDM (94 *S* : 6 *R*) were determined by chiral high-performance liquid chromatography (HPLC) analysis on a Lux Amylose-1 column, purchased from Phenomenex. The HPLC parameters employed included a mobile phase of hexane/isopropanol = 90:10, a flow rate of 1 mL min^–1^, a temperature of 25 °C and a detection wavelength of 210 nm. HPLC analysis was performed on a Waters Arc with a Waters 2998 PDA Wavelength UV Detector. All solvents employed were of HPLC grade.

## Results and Discussion

**Figure 3:**
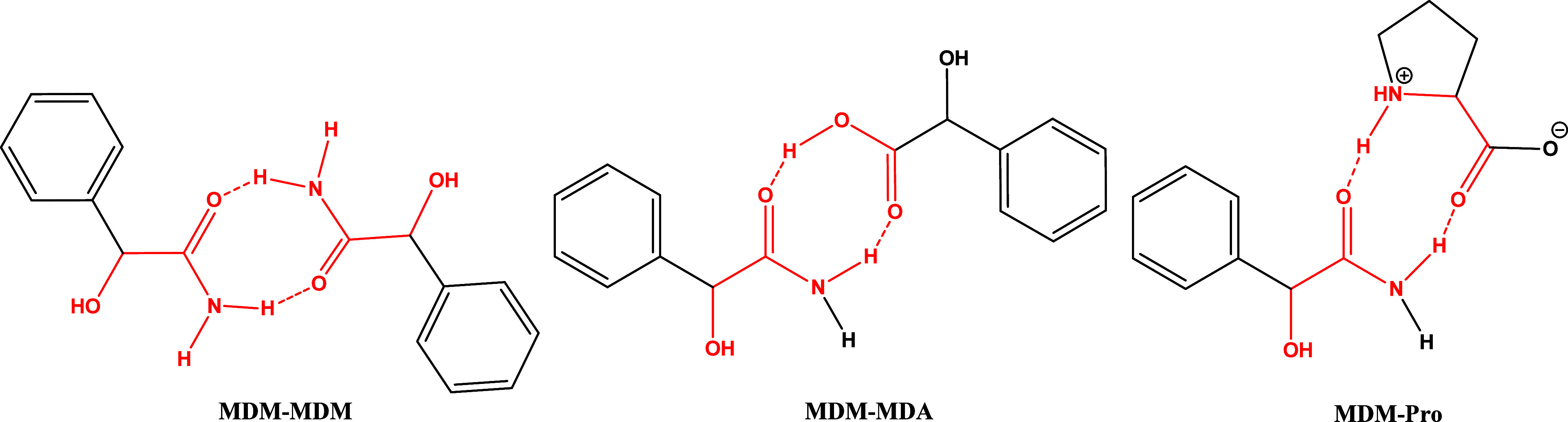
Expected hydrogen bond motifs in the MDM cocrystals.

Based on the molecular structures of mandelamide and both coformers, it was anticipated that the well-known amide–amide homosynthons and amide-acid heterosynthons would be observed in their crystal structures (Figure 3). A search of the CSD was undertaken to identify common supramolecular synthons for compounds containing a hydroxyl group in the α position to a primary or secondary amide functional group (shown in red in Figure 3). The *R*_2_^2^(8) homosynthon between two amides is commonly observed in 82 structures, 58 of which are single component crystals. Only one structure containing the amide-acid *R*_2_^2^(8) heterosynthon has been reported (Refcode NUGFAX^33^).

### Structures of Racemic and *S*-MDM

**Figure 4:**
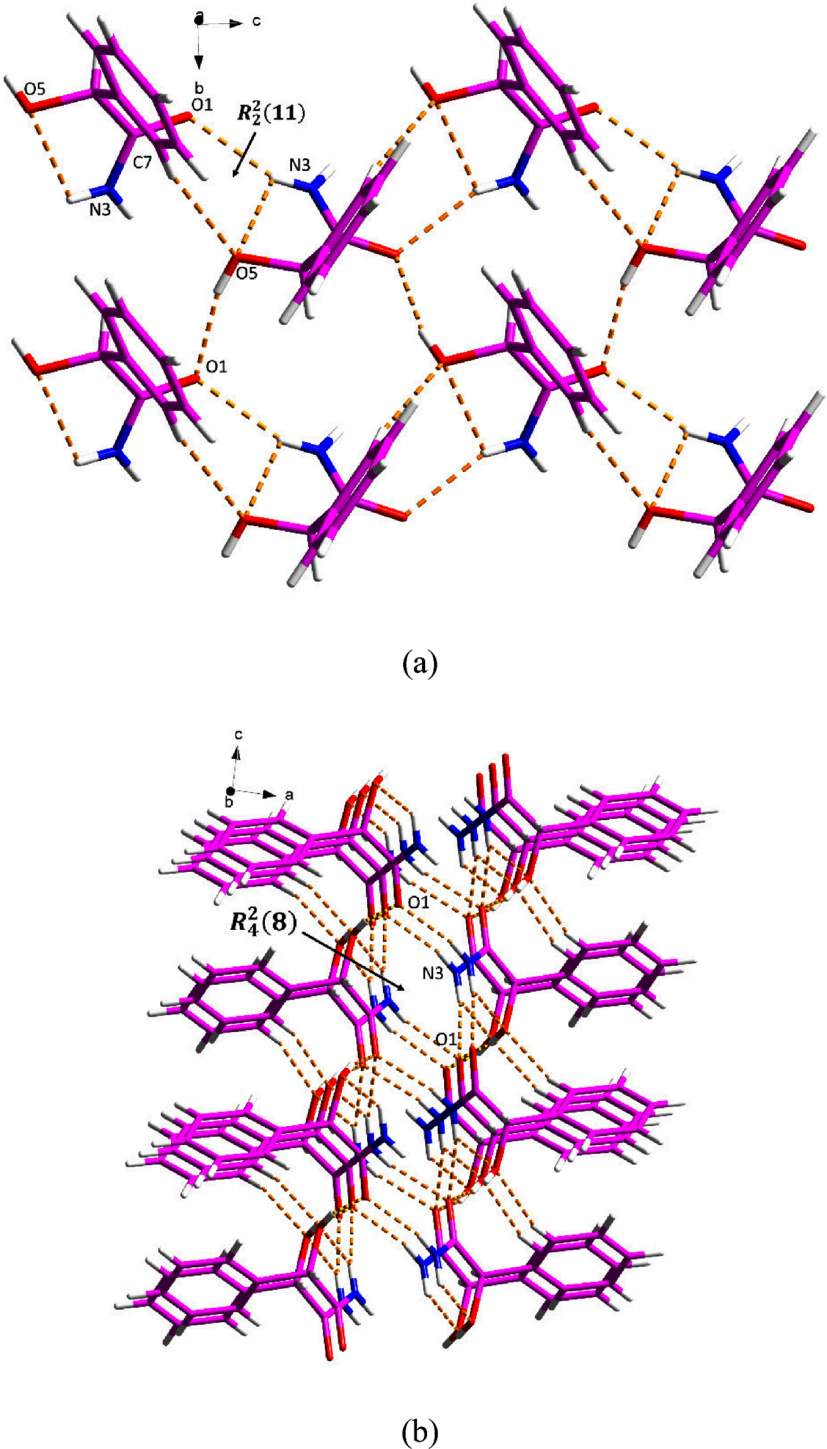
Hydrogen bonding in (±)-MDM: (a) along the *a* axis and (b) along the *b* axis.

**Figure 5:**
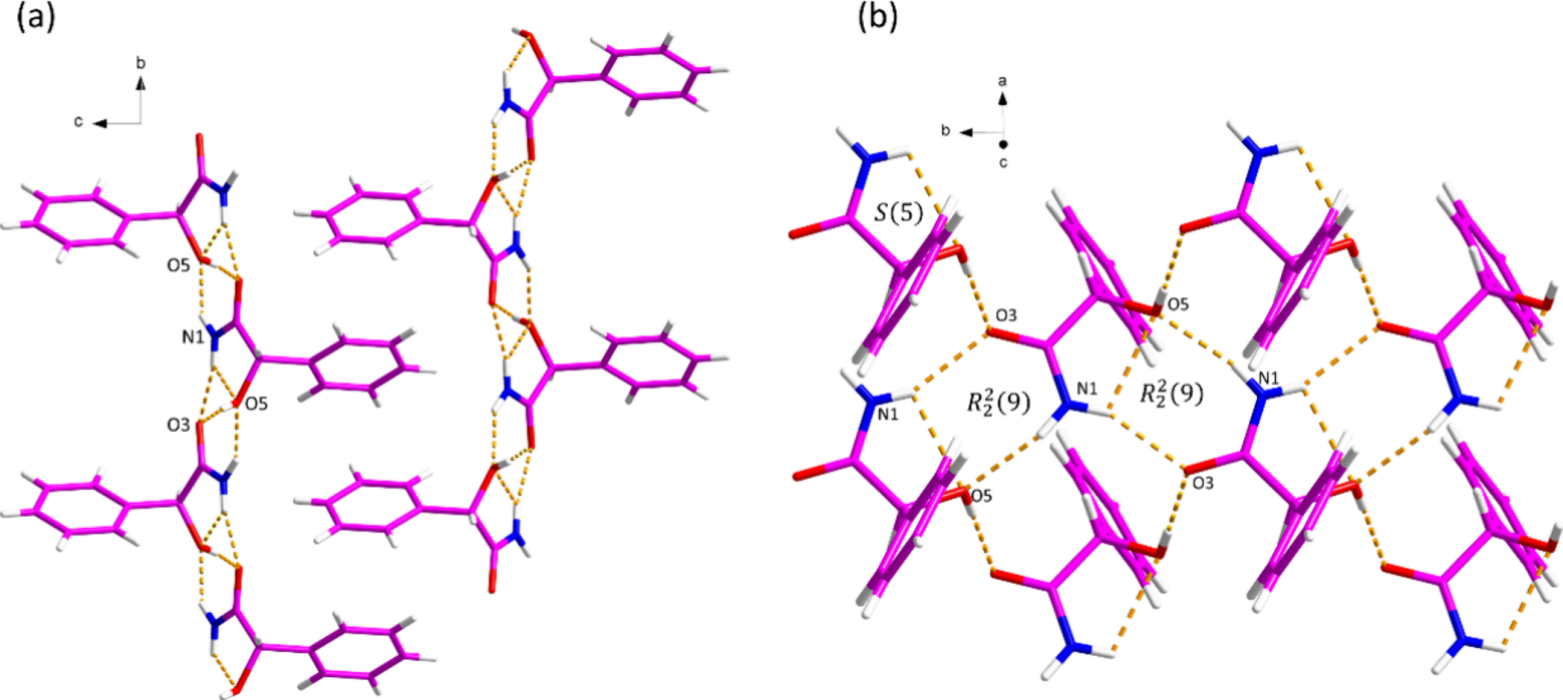
Hydrogen bonding in *S*-MDM.

Single crystals of racemic and enantiopure *S*-MDM were grown from THF and MeOH, respectively, and the structures determined as shown in Figure 4 and 5, respectively. The ellipsoid plots are shown in Figure S14. Hydrogen bonds and π–π interaction geometries are displayed in Tables S2 and S3, separately.

(±)-MDM crystallizes in the monoclinic *P*2_1_/*c* space group with *Z*′ = 1. As shown in Figure 4a, two (±)-MDM molecules formed a *R*_2_^2^(11) motif through the N–H···O and C–H···O hydrogen bonding. The hydrogen-bonded network is further extended by O–H···O hydrogen bonds between two (±)-MDM molecules. Along the *b* axis, an *R*_4_^2^(8) motif is created among four (±)-MDM molecules via N–H···O hydrogen bonding (Figure 4b).

*S*-MDM crystallizes in the *P2*_*1*_*2*_*1*_*2*_*1*_ space group with *Z*′ = 1. As shown in Figure 5, every two *S*-MDM molecules formed a *R*_2_^2^(9) dimer between the hydroxyl group and the amide group in a tail-to-tail manner through the N–H···O hydrogen bonding. The 3D hydrogen-bonded network is further stabilized by O–H···O hydrogen bonds between two *S*-MDM molecules.

Interestingly, during this study a third crystalline form of MDM was isolated from the solvent mixture of THF and toluene. Analysis of the SCXRD showed that this contained enantioenriched MDM (94 *S* : 6 *R*) which results in a very different structure relative to either the enantiopure or racemic forms. The chiral HPLC results on another crystal from the same batch are consistent with the structural analysis. (Figure S26). As shown in Figures S21 and S22, the crystal arrangement along the *b* axis in both the major and minor components of MDM (94 *S* : 6 *R*) exhibits similarity to the crystal packing observed in (±)-MDM, rather than the expected resemblance to *S*-MDM, despite the fact that *S*-MDM constitutes 94% of MDM (94 *S* : 6 *R*).

The single crystals of *S*-MDM were obtained from the synthesized *S*-MDM which contains 100% of *S*-MDM, while the formation of the MDM (94 *S* : 6 *R*) could be attributed to the commercial starting material being <100% *S*. According to the chiral HPLC analysis, the commercial *S*-MDM contained 96% *S*-MDM and 4% of *R*-MDM (Figure S24). PXRD analysis of the bulk material for (±)-MDM, *S*-MDM, and MDM (94 *S* : 6 *R*) match the theoretical PXRD based on the single crystal analysis, Figure S11. The formation of MDM (94 *S* : 6 *R*) may be rationalized either on the basis of solvent effects, since it was observed by crystallization from a THF/toluene mixture, or fast crystallization using the rotatory evaporator, which is a method that can produce new crystalline forms.^24^ To investigate whether MDM forms a solid solution, a 50:50 mixture of (±)-MDM and *R*-MDM was crystallized from methanol and analyzed by PXRD (Figure S11b). The peak at approximately 2θ = 19–20° matches all forms of MDM. It has low intensity broadening at lower 2θ (18–19°), which is the region where a peak is only observed in MDM (94 *S* : 6 *R*).

**Figure 6:**
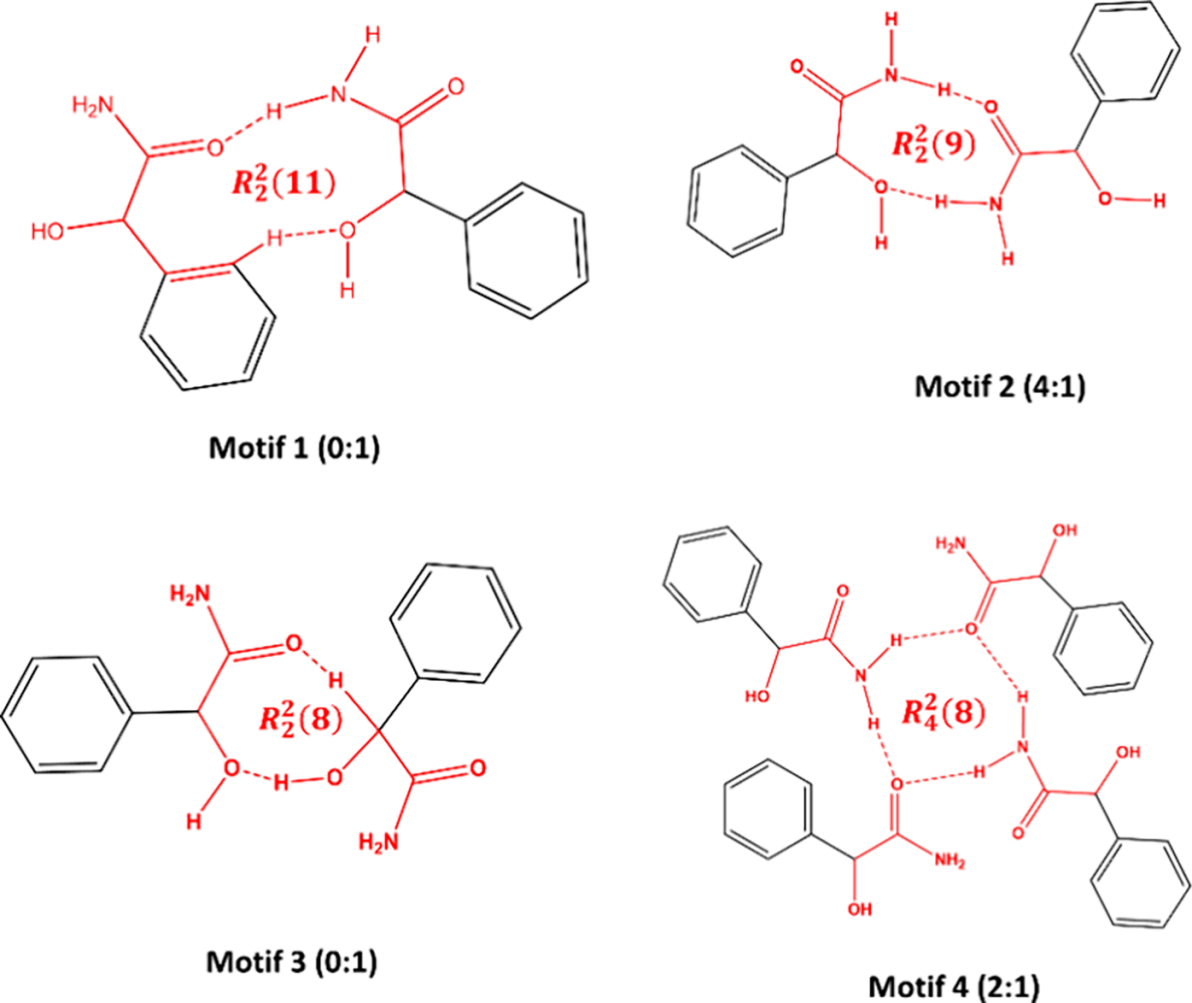
Types of motifs of MDM identified in this work. Numbers indicate occurrences in the CSD (left) and in this work (right).

The structural analysis results revealed that the expected *R*_2_^2^(8) motif between two MDM molecules is not present in any of the crystal structures of MDM. Instead, motifs 1–4 (Figure 6) are present in these three crystal structures. Motif 1 and 3 are not found in reported structures, while motif 2 was observed in four reported structures (Refcodes: VAFVIL,^34^ DEZKUR,^35^ NOLCOG,^36^ YENDEC^37^) based on the CSD search. In addition, motif 4, consisting of four MDM molecules in (±)-MDM and MDM (94 *S* : 6 *R*), can also be found in two reported structures (Refcodes: DEZLEC^35^ and YENDEC^37^).

The two main hydrogen-bonding functional groups in MDM are the amide and hydroxyl groups. As shown in Table S1 and Figure S3, the characteristic IR bands of the N–H and O–H stretches in (±)-MDM and MDM (94 *S* : 6 *R*) are both increased compared with those in *S*-MDM. In contrast, the stretching vibrations of C=O in these two solids display a decrease compared to *S*-MDM.

As shown in Figure S8, the melting point of (±)-MDM is 133–135 °C, which is in line with the reported data.^38^ DSC analysis of the MDM (94 *S* : 6 *R*) reveals its melting point is slight lower than that of *S*-MDM. In the book “Introduction to Stereochemistry”, Mislow examined the most common diastereomeric phase relationships that occur between two stereoisomers of similar substances.^39^ One out of the four scenarios could explain the thermal behavior of MDM (94 *S* : 6 *R*). In this case, introducing a small amount of impurity (i.e., *R*-MDM) can result in a decreased melting point compared to the pure component (*S*-MDM).

### Diastereomeric Cocrystal Pair of *S*-MDM and *S*/*R*-MDA

*S*-MDM-*S*-MDA and *S*-MDM-*R*-MDA cocrystals crystallized in the same space group (P*2*_*1*_*2*_*1*_*2*_*1*_) of the orthorhombic system and have similar unit cell parameters (Table 1). Hydrogen bonds and π–π interaction geometries are displayed in Tables S5 and S6, separately.

**Figure 7:**
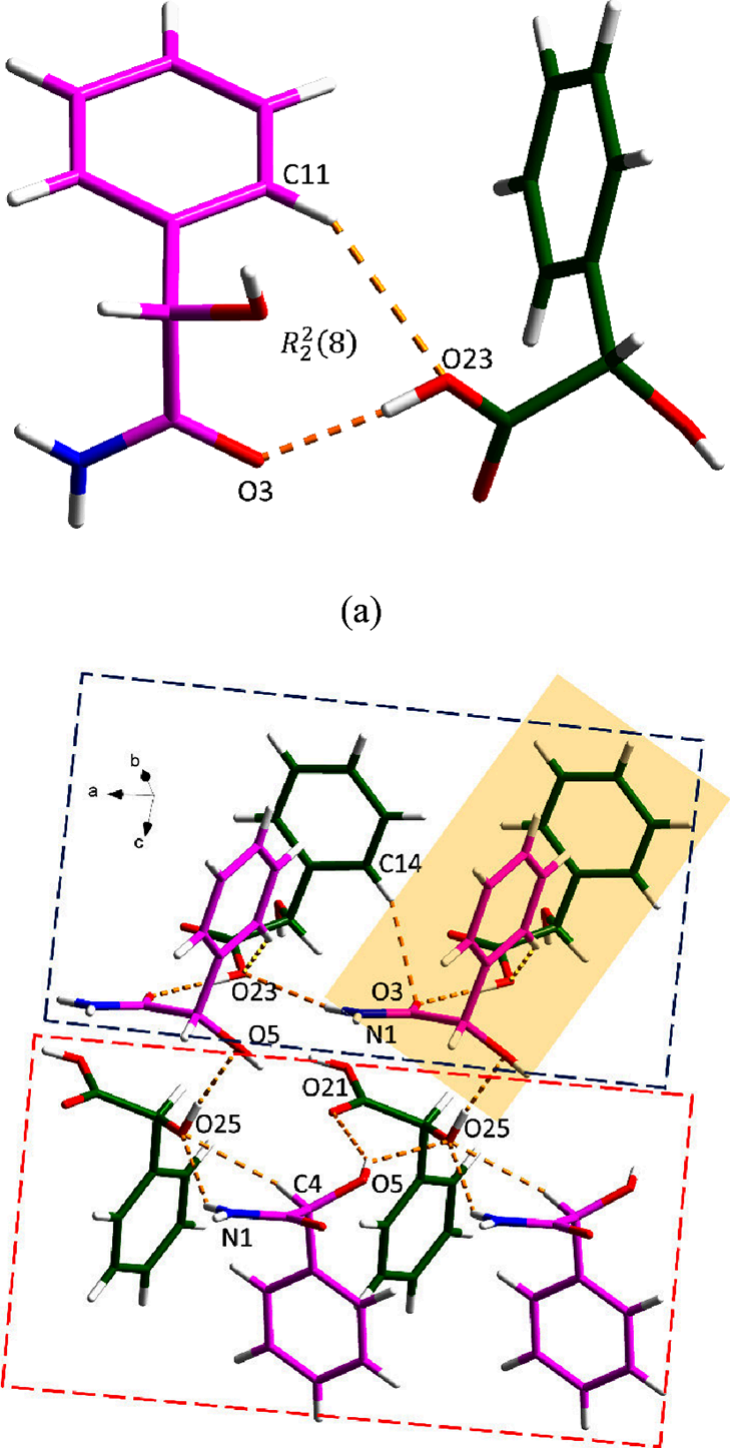
Hydrogen bonding in the *S*-MDM-*S*-MDA cocrystal: (a) asymmetric unit (pink is *S*-MDM and green is *S*-MDA) and (b) 3D hydrogen-bonded network.

*S*-MDM-*S*-MDA crystallizes with one *S*-MDM molecule and one *S*-MDA molecule in the asymmetric unit, Figure 7a. These two molecules are connected via C11–H11···O23 and O23–H23···O3 discrete hydrogen bonds, forming a *R*_2_^2^(8) motif. Two asymmetric units link through N1–H1A···O23 and C14–H14···O3 discrete hydrogen bonds, generating a four-molecule motif (top of Figure 7b). In the other four-molecule motif (bottom of Figure 7b), one *S*-MDM molecule and one *S*-MDA molecule interact through N1–H1B···O25 and C4–H4···O25 discrete hydrogen bonds, forming a similar four-molecule motif via O5–H5···O25 and O5–H5···O21 discrete hydrogen bonds. These two motifs are further assembled by an O25–H25···O5 hydrogen bond.

**Figure 8:**
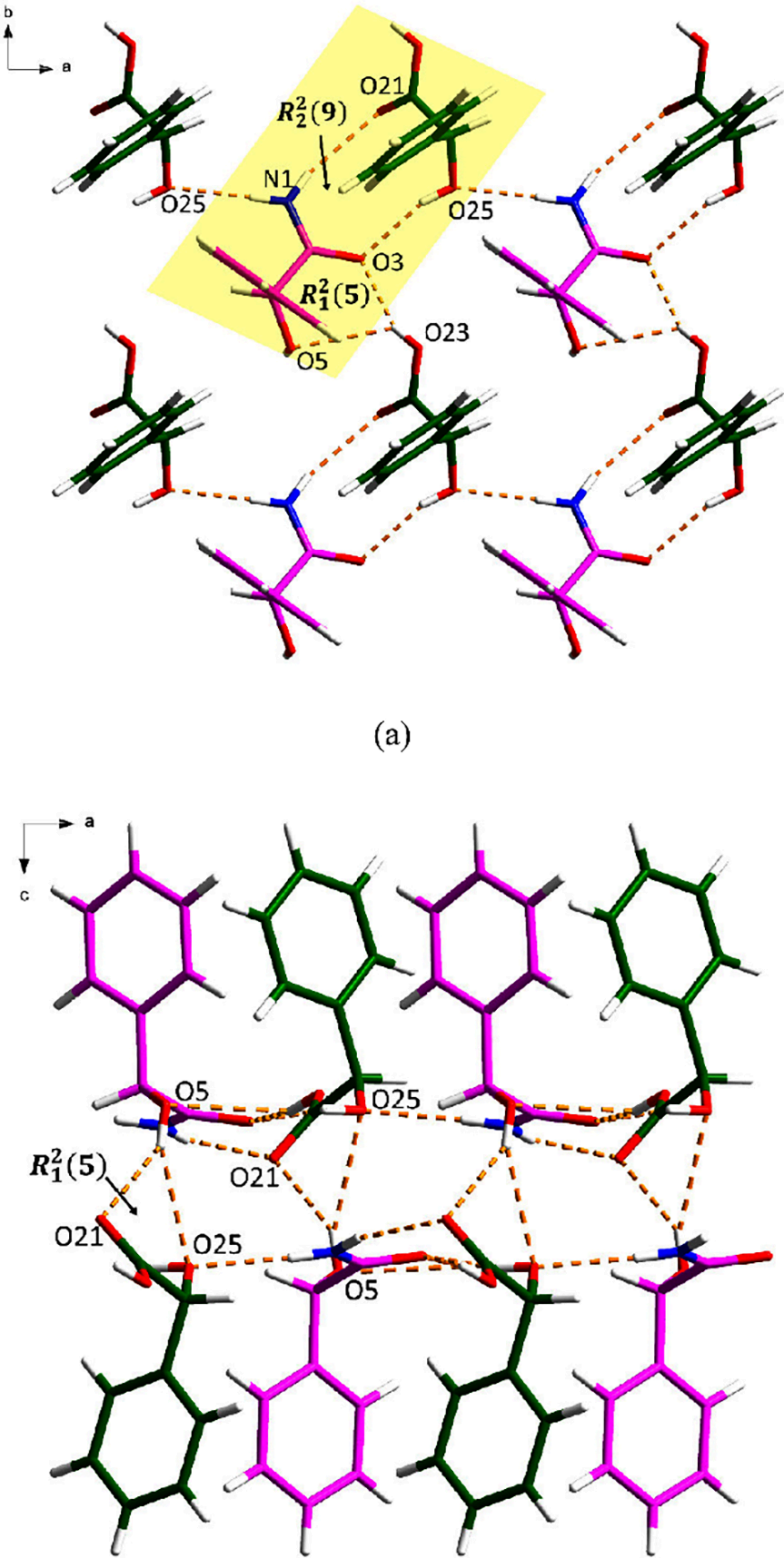
Hydrogen bonding in the S-MDM-R-MDA cocrystal (pink is *S*-MDM and green is *R*-MDA): (a) along the *a* axis, and (b) along the *b* axis.

The asymmetric unit of *S*-MDM-*R*-MDA contains one *S*-MDM molecule and one *R*-MDA molecule, which are connected via N1–H1A···O21 and O25–H25···O3 discrete hydrogen bonds, forming an *R*_2_^2^(9) motif. Along the *c* axis, the asymmetric unit links two adjacent units to extend the 3D structure of the cocrystal through O–H···O hydrogen bonds (forming an *R*_1_^2^(5) motif), and N–H···O hydrogen bond, respectively (Figure 8a). Additional hydrogen bonding between *S*-MDM and *R*-MDA molecules is observed in a tail-to-tail manner along the *b* axis, where an *R*_1_^2^(5) motif is created via O–H···O hydrogen bonds (Figure 8b).

The DSC data for the *S*-MDM-*S*-MDA and *S*-MDM-*R*-MDA cocrystals show single endothermic peaks at 85 and 81 °C, respectively, with the melting point of the cocrystals lying lower than those of the corresponding starting materials (Figure S9). As shown in Table S1 and Figures S4 and S5, the −NH_2_, −OH, and C=O bands of *S*-MDM exhibit shifts in both cocrystals. All the observed differences indicated that those three moieties are involved in the formation of hydrogen bonds in the different cocrystals. As shown in Figure S12 the PXRD patterns for both *S*-MDM-*S*-MDA and *S*-MDM-*R*-MDA cocrystals match with the simulated patterns extracted from the SCXRD analysis, indicating these cocrystals can be reproduced in bulk quantities by the LAG method. The products were the same irrespective of the source of *S*-MDM (synthesized or commercial) used in the experiments.

### Diastereomeric Cocrystal Pair of *S*-MDM and l/d-Pro

**Figure 9:**
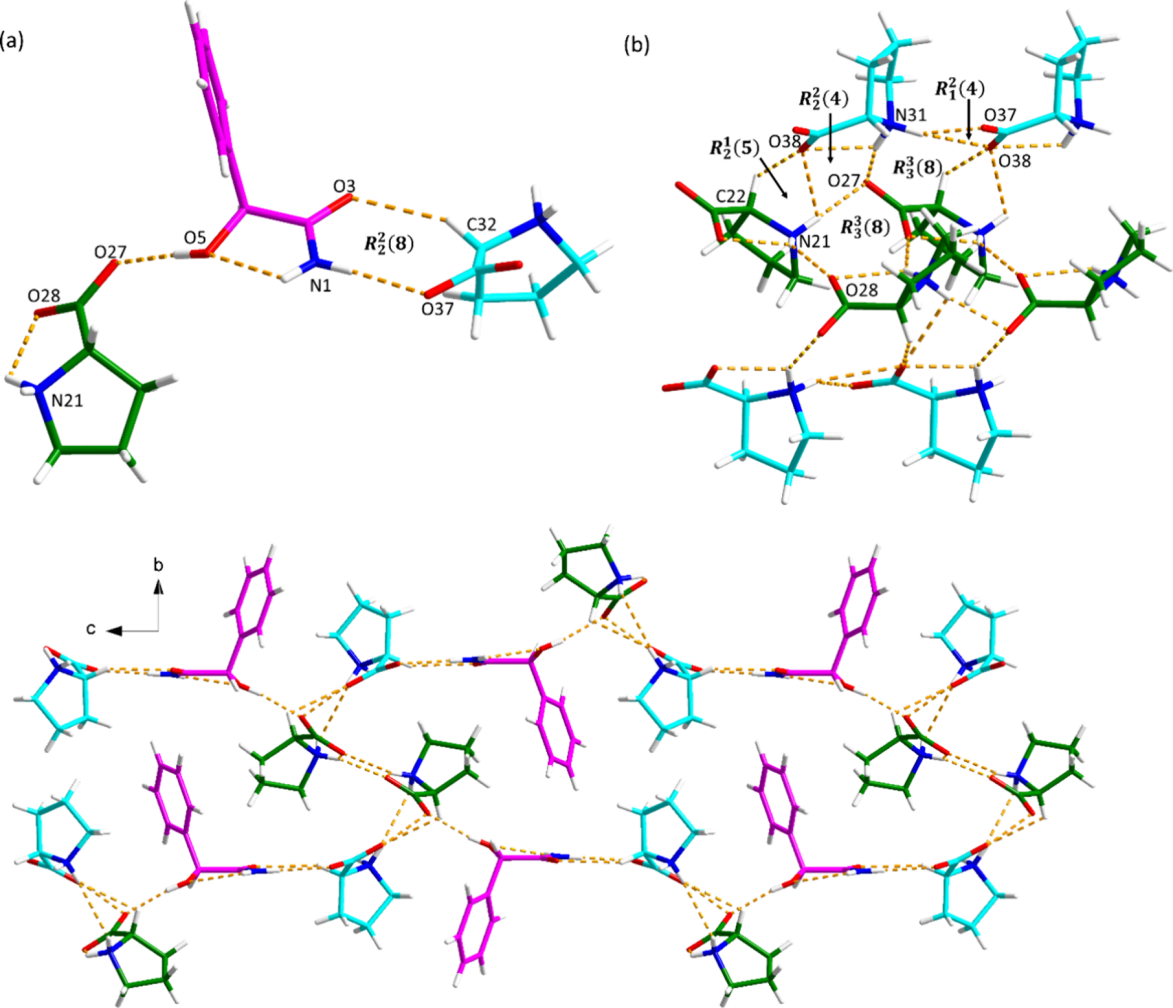
Hydrogen bonding in the *S*-MDM-l-Pro cocrystal (pink is *S*-MDM, green is l-Pro 1, and blue is l-Pro 2): (a) hydrogen bonding between *S*-MDM and two l-Pro molecules, (b) hydrogen bonding between l-Pro molecules, and (c) 3D hydrogen-bonded network along the *a* axis. One of the disordered carbon atom conformations of l-Pro 2 has been omitted for clarity.

A stoichiometrically diverse diastereomeric cocrystal system between *S*-MDM and l/d-Pro was obtained. Hydrogen bonds and π–π interaction geometries are displayed in Tables S7 and S8, respectively. *S*-MDM-l-Pro cocrystallized in the orthorhombic *P*2_1_2_1_2_1_ space group with one *S*-MDM and two l-Pro molecules in the asymmetric unit. As shown in Figure 9a, *S*-MDM links l-Pro 1 through O5–H5···O27 hydrogen bond and connects l-Pro 2 via N–H···O and C–H···O hydrogen bonds, forming an *R*_2_^2^(8) motif. *R*_1_^2^(4), *R*_2_^1^(5), and *R*_3_^3^(8) motifs between l-Pro molecules interlink the chain (Figure 9b), stabilizing the 3D hydrogen-bonded network of S-MDM-l-Pro cocrystal along the *a* axis (Figure 9c).

**Figure 10:**
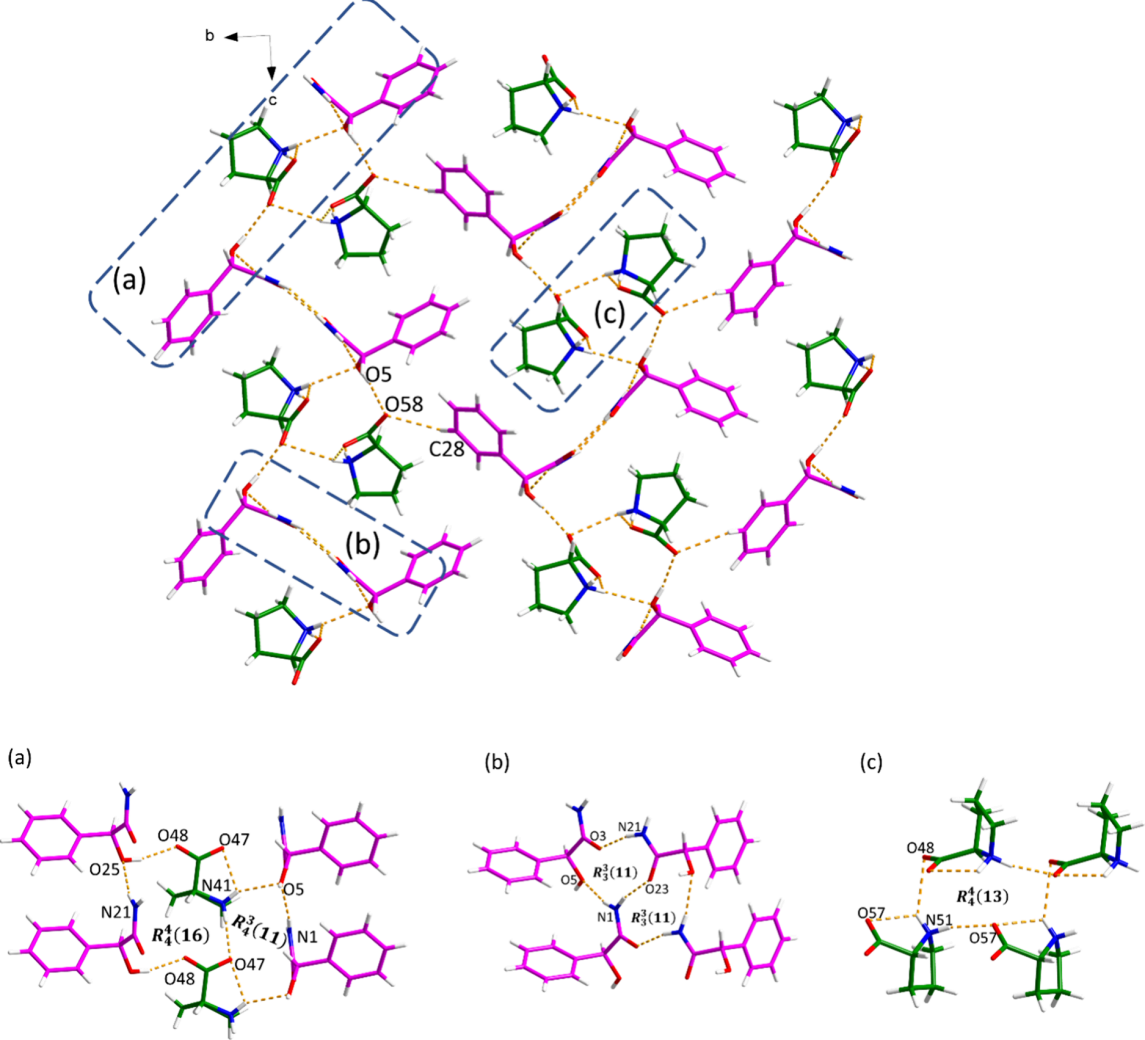
Hydrogen bonding in the *S*-MDM-d-Pro cocrystal. The minor disordered component of d-Pro has been omitted for clarity.

The *S*-MDM-d-Pro cocrystal crystallizes in the monoclinic space group P*2*_*1*_ and the asymmetric unit consists of two *S*-MDM molecules and two d-Pro molecules (Z′ = 2). As shown in Figure 10, two *S*-MDM molecules and two d-Pro molecules can be regarded as the crystal packing building block, where *R*_4_^4^(16) motif and *R*_4_^3^(11) motif are created among four *S*-MDM molecules and two d-Pro molecules via N–H···O and O–H···O hydrogen bonds. An *R*_4_^4^(13) motif between four d-Pro molecules is also observed in this building block through N–H···O hydrogen bonding. The 3D hydrogen-bonded network is extended by connecting different building blocks through O5–H5···O58 and C28–H28···O58 hydrogen bonds. Meanwhile, N–H···O hydrogen bonds between four *S*-MDA molecules also contribute to the stabilization of the crystal structure, forming two *R*_3_^3^(11) motifs.

Adifference of melting point between the *S*-MDM-l-Pro and *S*-MDM-d-Pro cocrystals can be observed from the DSC plots (Figure S10). The *S*-MDM-l-Pro cocrystal shows a single endothermic peak at 208 °C and the melting point of *S*-MDM-d-Pro cocrystal is 166 °C. Both of these are in between that of the individual components. The IR data show differences in the ν_N–H_, ν_O–H_, and ν_C=O_, indicating reconstruction of hydrogen bond networks in those solids and the formation of new crystalline solids (Figures S6 and S7). The experimental PXRD patterns of S-MDM-l-Pro and S-MDM-d-Pro cocrystals were found to compare well with the simulated PXRD patterns obtained from the SCXRD data (Figure S13). The different sources of *S*-MDM used in the cocrystallization experiments did not influence the products obtained.

### Analysis of Diastereomeric Cocrystal Pairs of *S*-MDM

A 2014 CSD search of the existing enantiospecific and diastereomeric cocrystals demonstrated that among 44 multicomponent structures containing two optically active compounds, 38 (86%) systems behave enantiospecifically.^40^ This reveals that even a small change in the structure of the cocrystallizing component, such as a change in absolute and/or relative stereochemistry, can lead to changes in secondary interactions and steric effects, ultimately changing the outcome of cocrystal formation.^40−44^ Flood et al. explored the formation of enantiospecific and diastereomeric cocrystals by employing crystal structure prediction and molecular simulations, indicating that despite the similarity in the predicted hypothetical crystal structure and hydrogen-bonding geometries, variations in aromatic interactions and lattice energy were instrumental in favoring the formation of an enantiospecific cocrystal instead of a diastereomeric cocrystal pair.^45^ Therefore, for the formation of a diastereomeric cocrystal pair, more changes in the hydrogen bonding network and molecular arrangement are required in order to reduce the influence of the secondary interactions and steric effects to the total cocrystal stabilization energy.^40^

**Figure 11:**
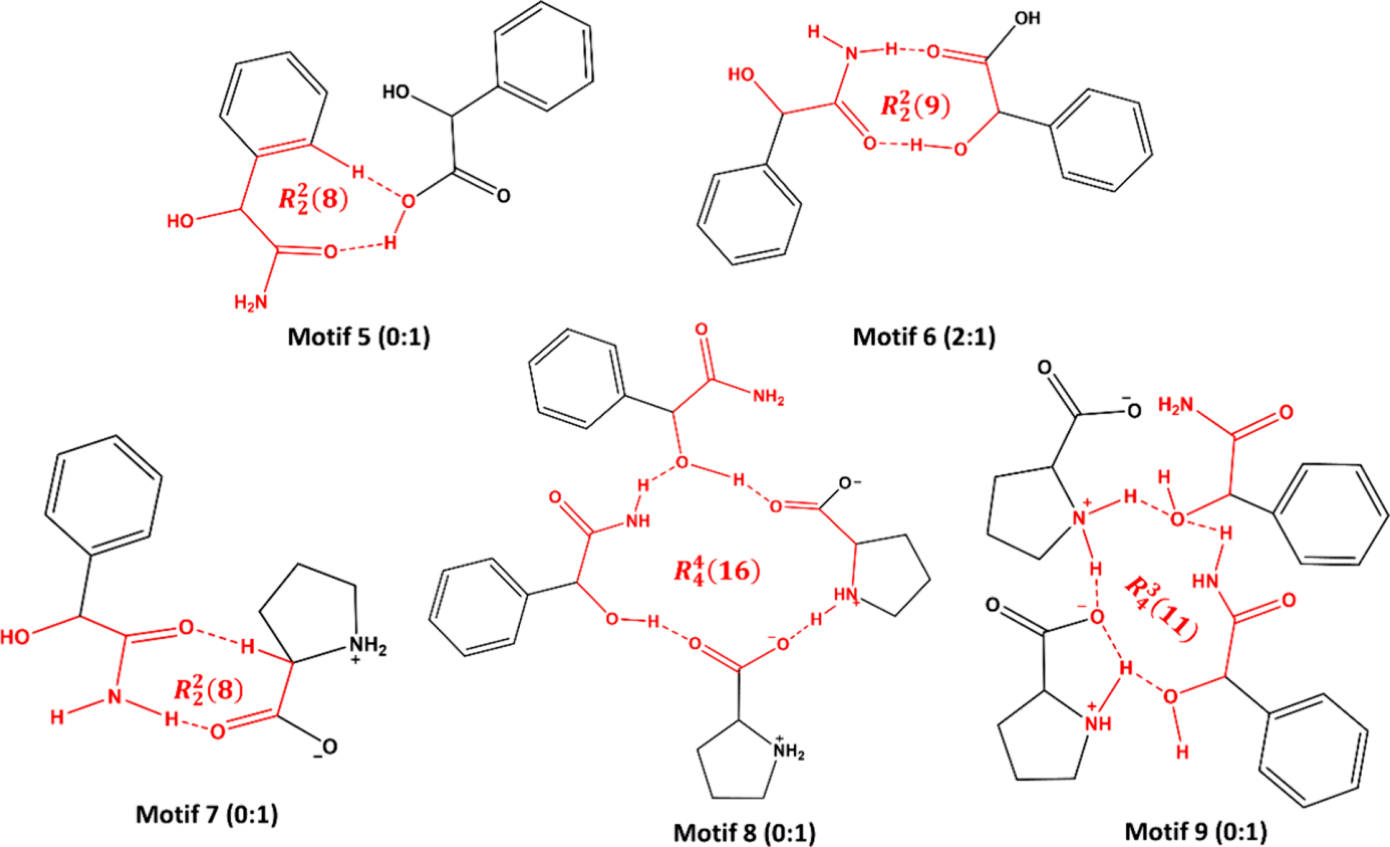
Types of motifs of *S*-MDM with coformers identified in this work. Numbers indicate occurrences in the CSD (left) and in this work (right).

As mentioned earlier, diastereomeric cocrystals of *S*-MDM with *S*/*R*-MDA have similar crystallographic data, and the stoichiometric ratio between *S*-MDM and the coformers are the same. However, the hydrogen bonding between the two components in these cocrystals differ significantly. As shown in Figure 11, binary level hydrogen-bonding motifs are present in *S*-MDM-*S*-MDA (motif 5) and *S*-MDM-*R*-MDA cocrystals (motif 6), respectively. For the *S*-MDM-*S*-MDA cocrystal, only the hydroxyl group from the carboxyl group of *S*-MDA, serving as both hydrogen-bonding donor and acceptor, is engaged in the hydrogen bond formation, while both the oxygen atom of the carbonyl group and a hydrogen atom (H11) from the benzene ring of *S*-MDM are involved in the hydrogen bond construction. In contrast, for the *S*-MDM-*R*-MDA cocrystal, hydrogen bonding occurs between the carbonyl oxygen atom and the hydroxyl group of *R*-MDA and the amide group of *S*-MDM. Motif 5 is not found in any structures through the CSD search, whereas motif 6 was presented in two reported (Refcodes: VASWOC^46^ and ZZZRJG01^47^). These orientationally restrictive interaction motifs determine the formation of diastereomeric cocrystal pairs between *S*-MDM and *S*/*R*-MDA.^48^ Moreover, the different contacts in these two cocrystals can be visualized by their 2D fingerprint plots (Figure S23a and Table S9). Hydrogen bonding in the *S*-MDM-*S*-MDA cocrystal constitute a bigger proportion compared with those in *S*-MDM-*R*-MDA cocrystal, while in contrast, van der Waals interactions account for a larger percentage in the *S*-MDM-*R*-MDA cocrystal. These significant differences lead to the remarkable changes in the crystal packing for this diastereomeric pair.

Compared to the *S*-MDM-*S*/*R*-MDA diastereomeric cocrystal pair, the differences between the *S*-MDM-l-Pro and *S*-MDM-d-Pro cocrystals are more significant. Apart from the dissimilar motifs (motif 7 from *S*-MDM-l-Pro, motifs 8 and 9 from *S*-MDM-d-Pro) resulting from different functional groups in two cocrystals (Figures 9 and 10) and their distinct 2D fingerprint plots and corresponding contact contributions (Figure S23b and Table S9), the primary factor that overcame the obstacle of stabilization free energy for cocrystal formation is the varying stoichiometric ratios of *S*-MDM and l/d-Pro. This is similar to the recent report by Leyssens and co-workers for l-Pro with mandelic acid.^20^

Given the different outcomes in terms of stoichiometry when using the diastereomeric pairs of S-MDM with either MDA or proline, a series of screening experiments were conducted with S-MDM and *S*/*R*-MDA and l/d-Pro in 1:1, 1:2, and 2:1 ratios. Based on the PXRD analysis, the product 1:1 ratio is of high purity without the existence of the diffraction peaks from either *S*-MDM or *S*/*R*-MDA. The PXRD pattern of the new phase of S-MDM with l-Pro in a 1:2 ratio was obtained, while for the 1:1 and 2:1 ratios, excess S-MDM was present as well as the 1:2 product. For the d-Pro system, new diffraction peaks of S-MDM-d-Pro were found using a 1:1 ratio. Excess S-MDM was detected when a 2:1 ratio was used and excess d-Pro found using a 1:2 ratio. These grinding experiment results are in line with the solution crystallization results.

To demonstrate the potential of the MDM as a cocrystal system for chiral resolution, a series of slurry experiments involving (±)-MDM and l-Pro in molar ratios ranging from 1:1 to 1:5 were undertaken (Table S10). The PXRD results revealed that at high proportions of l-Pro, particularly 1:4 and 1:5 ratios, the *R*-MDM:l-Pro (or *S*-MDM-d-Pro) cocrystals were not detected. Due to challenges in the determination of the enantiopurity of proline, the resolution experiment was undertaken using (±)-MDM and l-Pro as a proof of concept. Thus, a sample of (±)-MDM and l-Pro (in 1:3–1:5 molar ratios) was slurried in MeOH for 3 d. Separation of the solid from the liquid phase and analysis of each component revealed that the solid consisted predominantly of *S*-MDM-l-Pro by PXRD. Notably, the enantiopurity of *S*-MDM in the solid phase with 1:5 ratio is 96.1%ee, confirming the chiral resolution is possible through this cocrystal system (Figure S27). Further investigations are underway to explore the potential of MDM for chiral resolution through cocrystallization.

## Conclusions

In summary, the crystal structures of (±)-MDM, *S*-MDM and MDM (94 *S* : 6 *R*) were identified and fully characterized in this work. Additionally, this study reports the synthesis and characterization of two novel diastereomeric cocrystal pairs of *S*-MDM with both enantiomers of mandelic acid (*S*-MDM-*S*-MDA and *S*-MDM-*R*-MDA) and proline (*S*-MDM-l-Pro and *S*-MDM-d-Pro). The *S*-MDM-*S*-MDA and *S*-MDM-*R*-MDA cocrystals have similar unit cell parameters and the same stoichiometric ratio (1:1), yet a significantly different hydrogen bonding between the two coformers plays a critical structure determining role. The formation of *S*-MDM-l-Pro and *S*-MDM-d-Pro diastereomeric cocrystals proceeds with different stoichiometries, similar to a recent report of proline with mandelic acid,^20^ although the structure determining features are very different. The feasibility of utilizing MDM and l-Pro as a cocrystal system for chiral resolution was explored. This work revealed that *S*-MDM can be effectively resolved by cocrystallization with l-proline.

## Supporting Information Available

Synthesis of (±)-MDM, *S*-MDM, NMR spectra, FT-IR spectra, DSC plots, PXRD patterns, ellipsoid plots, hydrogen bonding in MDM (94 *S* : 6 *R*), chiral HPLC results, hydrogen-bonding data, Hirshfield analysis results, summary of the slurry experiments and chiral resolution results, summary of the reported cocrystals of mandelic acid and chiral coformers (PDF)

## References

[ref1] Inaki M.; Liu J.; Matsuno K. Cell chirality: its origin and roles in left–right asymmetric development. Philos. Trans. R. Soc. London B Biol. Sci. 2016, 371 (1710), 20150403 10.1098/rstb.2015.0403. 27821533 PMC5104503

[ref2] Liu Y.; Xiao J.; Koo J.; Yan B. Chirality-driven topological electronic structure of DNA-like materials. Nat. Mater. 2021, 20, 638–644. 10.1038/s41563-021-00924-5. 33558719 PMC7610709

[ref3] Brandt J. R.; Salerno F.; Fuchter M. J. The added value of small-molecule chirality in technological applications. Nat. Rev. Chem. 2017, 1, 0045 10.1038/s41570-017-0045.

[ref4] Zhou Y.; Wu S.; Zhou H.; Huang H.; Zhao J.; Deng Y.; Wang H.; Yang Y.; Yang J.; Luo L. Chiral pharmaceuticals: Environment sources, potential human health impacts, remediation technologies and future perspective. Environ. Int. 2018, 121, 523–537. 10.1016/j.envint.2018.09.041. 30292145

[ref5] McConathy J.; Owens M. J. Stereochemistry in Drug Action. Primary Care Companion CNS Psychiatry 2003, 5 (2), 70–73. 10.4088/PCC.v05n0202. PMC353039 15156233

[ref6] Blessington B.; Beiraghi A. Study of the stereochemistry of ethambutol using chiral liquid chromatography and synthesis. J. Chromatogr. A 1990, 522, 195–203. 10.1016/0021-9673(90)85189-3.

[ref7] Brooks W. H.; Guida W. C.; Daniel K. G. The Significance of Chirality in Drug Design and Development. Curr. Top. Med. Chem. 2011, 11 (7), 760–770. 10.2174/156802611795165098. 21291399 PMC5765859

[ref8] Ault A. The Nobel Prize in Chemistry for 2001. J. Chem. Educ. 2002, 79 (5), 572 10.1021/ed079p572.

[ref9] Nguyen L. A.; He H.; Pham-Huy C. Chiral Drugs: An Overview. Int. J. Biol. Sci. 2006, 2 (2), 85–100. 10.59566/IJBS.2006.2085. PMC3614593 23674971

[ref10] Lee H. L.; Hung Y. L.; Amin A.; Pratama D. E.; Lee T. Green and Strategic Approach for Chiral Resolution by Diastereomeric Salt Formation: The Study of Racemic Ibuprofen. Ind. Eng. Chem. Res. 2023, 62 (4), 1946–1957. 10.1021/acs.iecr.2c04290.

[ref11] Yang L.-C.; Deng H.; Renata H. Recent Progress and Developments in Chemoenzymatic and Biocatalytic Dynamic Kinetic Resolution. Org. Process Res. Dev. 2022, 26 (7), 1925–1943. 10.1021/acs.oprd.1c00463.

[ref12] Pinto M. M. M.; Fernandes C.; Tiritan M. E. Chiral Separations in Preparative Scale: A Medicinal Chemistry Point of View. Molecules. 2020, 25 (8), 1931 10.3390/molecules25081931. 32326326 PMC7221958

[ref13] Pawar N.; Saha A.; Nandan N.; Parambil J. V. Solution Cocrystallization: A Scalable Approach for Cocrystal Production. Crystals 2021, 11 (3), 303 10.3390/cryst11030303.

[ref14] Springuel G.; Leyssens T. Innovative Chiral Resolution Using Enantiospecific Co-Crystallization in Solution. Cryst. Growth Des. 2012, 12, 3374–3378. 10.1021/cg300307z.

[ref15] Buol X.; Garrido C. C.; Robeyns K.; Tumanov N.; Collard L.; Wouters J.; Leyssens T. Chiral Resolution of Mandelic Acid through Preferential Cocrystallization with Nefiracetam. Cryst. Growth Des. 2020, 20, 7979–7988. 10.1021/acs.cgd.0c01236.

[ref16] Shemchuk O.; Song L.; Tumanov N.; Wouters J.; Braga D.; Grepioni F.; Leyssens T. Chiral Resolution of RS-Oxiracetam upon Cocrystallization with Pharmaceutically Acceptable Inorganic Salts. Cryst. Growth Des. 2020, 20, 2602 10.1021/acs.cgd.9b01725.

[ref17] Harmsen B.; Leyssens T. Dual-Drug Chiral Resolution: Enantiospecific Cocrystallization of (*S*)-Ibuprofen Using Levetiracetam. Cryst. Growth Des. 2018, 18 (1), 441–448. 10.1021/acs.cgd.7b01431.

[ref18] Shemchuk O.; Grepioni F.; Leyssens T.; Braga D. Chiral Resolution via Cocrystallization with Inorganic Salts. Isr. J. Chem. 2021, 61, 563–572. 10.1002/ijch.202100049.

[ref19] Sánchez-Guadarrama O.; Mendoza-Navarro F.; Cedillo-Cruz A.; Jung-Cook H.; Arenas-García J. I.; Delgado-Díaz A.; Herrera-Ruiz D.; Morales-Rojas H.; Höpfl H. Chiral Resolution of *RS*-Praziquantel via Diastereomeric Co-Crystal Pair Formation with L-Malic Acid. Cryst. Growth Des. 2016, 16 (1), 307–314. 10.1021/acs.cgd.5b01254.

[ref20] Zhou F.; Collard L.; Robeyns K.; Leyssens T.; Shemchuk O. L-Proline, a resolution agent able to target both enantiomers of mandelic acid: an exciting case of stoichiometry controlled chiral resolution. Chem. Commun. 2022, 58, 8560–8563. 10.1039/D2CC02942A. 35815867

[ref21] Groom C. R.; Bruno I. J.; Lightfoot M. P.; Ward S. C. The Cambridge Structural Database. Acta Crystallogr., Sect. B: Struct. Sci., Cryst. Eng. Mater. 2016, 72, 171–179. 10.1107/S2052520616003954. PMC4822653 27048719

[ref22] Stokes M. E.; Surman M. D.; Calvo V.; Surguladze D.; Li A.-H.; Gasparek J.; Betzenhauser M.; Zhu G.; Du H.; Rigby A. C.; Mulvihill M. J. Optimization of a Novel Mandelamide-Derived Pyrrolopyrimidine Series of PERK Inhibitors. Pharmaceutics 2022, 14 (10), 2233 10.3390/pharmaceutics14102233. 36297668 PMC9611727

[ref23] Lloyd-Jones G. C.; Wall P. D.; Slaughter J. L.; Parker A. J.; Laffan D. P. Enantioselective homoallyl-cyclopropanation of dibenzylideneacetone by modified allylindium halide reagents—rapid access to enantioenriched 1-styryl-norcarene. Tetrahedron 2006, 62 (49), 11402–11412. 10.1016/j.tet.2006.05.003.

[ref24] Bag P. P.; Reddy C. M. Screening and Selective Preparation of Polymorphs by Fast Evaporation Method: A Case Study of Aspirin, Anthranilic Acid, and Niflumic Acid. Cryst. Growth Des. 2012, 12 (6), 2740–2743. 10.1021/cg300404r.

[ref25] Sinha A. S.; Rao Khandavilli U. B.; O’Connor E. L.; Deadman B. J.; Maguire A. R.; Lawrence S. E. Novel co-crystals of the nutraceutical sinapic acid. CrystEngComm 2015, 17 (26), 4832–4841. 10.1039/C5CE00777A.

[ref26] Sheldrick G. M. Crystal structure refinement with SHELXL. Acta Crystallogr., Sect. C: Struct. Chem. 2015, 71 (Pt 1), 3–8. 10.1107/S2053229614024218. 25567568 PMC4294323

[ref27] APEX2 v2009.3–0. Bruker AXS Inc. 2009.

[ref28] Xu J.; Huang Y.; Ruan S.; Chi Z.; Qin K.; Cai B.; Cai T. Cocrystals of isoliquiritigenin with enhanced pharmacokinetic performance. CrystEngComm 2016, 18 (45), 8776–8786. 10.1039/C6CE01809B.

[ref29] Spek A. L. Structure validation in chemical crystallography. Acta Crystallogr., Sect. D: Biol. Crystallogr. 2009, 65, 148–155. 10.1107/S090744490804362X. 19171970 PMC2631630

[ref30] Huang S.; Xu J.; Peng Y.; Guo M.; Cai T. Facile Tuning of the Photoluminescence and Dissolution Properties of Phloretin through Cocrystallization. Cryst. Growth Des. 2019, 19 (12), 6837–6844. 10.1021/acs.cgd.9b01111.

[ref31] Spackman P. R.; Turner M. J.; McKinnon J. J.; Wolff S. K.; Grimwood D. J.; Jayatilaka D.; Spackman M. A. CrystalExplorer: a program for Hirshfeld surface analysis, visualization and quantitative analysis of molecular crystals. J. Appl. Crystallogr. 2021, 54 (Pt 3), 1006–1011. 10.1107/S1600576721002910. 34188619 PMC8202033

[ref32] Bruno I. J.; Cole J. C.; Edgington P. R.; Kessler M.; Macrae C. F.; McCabe P.; Pearson J.; Taylor R. New software for searching the Cambridge Structural Database and visualizing crystal structures. Acta Crystallogr. 2002, B58, 389–397. 10.1107/S0108768102003324. 12037360

[ref33] Almog J.; Rozin R.; Klein A.; Shamuilov-Levinton G.; Cohen S. The reaction between phloroglucinol and vic polycarbonyl compounds: extension and mechanistic elucidation of Kim’s synthesis for bipolarofacial bowl-shaped compounds. Tetrahedron 2009, 65 (38), 7954–7962. 10.1016/j.tet.2009.07.056.

[ref34] Smit J. P., CCDC 2109758. Experimental Crystal Structure Determination 2021, DOI: 10.5517/ccdc.csd.cc28tcq6.

[ref35] Pinter E. N.; Cantrell L. S.; Day G. M.; Wheeler K. A. Pasteur’s tartaramide/malamide quasiracemates: new entries and departures from near inversion symmetry. CrystEngComm 2018, 20, 4213–4220. 10.1039/C8CE00791H.

[ref36] Gawroński J.; Gawrońska K.; Skowronek P.; Rychlewska U.; Warżajtis B.; Rychlewski J.; Hoffmann M.; Szarecka A. Factors Affecting Conformation of (R,R)-Tartaric Acid Ester, Amide and Nitrile Derivatives. X-Ray Diffraction, Circular Dichroism, Nuclear Magnetic Resonance and Ab Initio Studies. Tetrahedron 1997, 53 (17), 6113–6144. 10.1016/S0040-4020(97)00271-8.

[ref37] Janiak A.; Rychlewska U.; Kwit M.; Stępień U.; Gawrońska K.; Gawroński J. From Single Molecule to Crystal: Mapping Out the Conformations of Tartaric Acids and Their Derivatives. ChemPhysChem 2012, 13, 1500–1506. 10.1002/cphc.201200033. 22416051

[ref38] Kanda T.; Naraoka A.; Naka H. Catalytic Transfer Hydration of Cyanohydrins to α-Hydroxyamides. J. Am. Chem. Soc. 2019, 141 (2), 825–830. 10.1021/jacs.8b12877. 30590921

[ref39] Mislow K. Introduction to Stereochemistry. ed.; W. A. Benjamin Inc.: New York, Amsterdam, 1966.

[ref40] Springuel G.; Robeyns K.; Norberg B.; Wouters J.; Leyssens T. Cocrystal Formation between Chiral Compounds: How Cocrystals Differ from Salts. Cryst. Growth Des. 2014, 14 (8), 3996–4004. 10.1021/cg500588t.

[ref41] Taylor C. R.; Day G. M. Evaluating the Energetic Driving Force for Cocrystal Formation. Cryst. Growth Des. 2018, 18, 892–904. 10.1021/acs.cgd.7b01375. PMC5806084 29445316

[ref42] Huang S.; Cheemarla V. K. R.; Tiana D.; Lawrence S. E. Experimental and Theoretical Investigation of Hydrogen-Bonding Interactions in Cocrystals of Sulfaguanidine. Cryst. Growth Des. 2023, 23 (4), 2306–2320. 10.1021/acs.cgd.2c01337. PMC10080660 37038403

[ref43] Arunan E.; Desiraju G. R.; Klein R. A.; Sadlej J.; Scheiner S.; Alkorta I.; Clary D. C.; Crabtree R. H.; Dannenberg J. J.; Hobza P.; Kjaergaard H. G.; Legon A. C.; Mennucci B.; Nesbitt a. D. J. Definition of the hydrogen bond (IUPAC Recommendations 2011). Pure Appl. Chem. 2011, 83 (8), 1637–1641. 10.1351/PAC-REC-10-01-02.

[ref44] Shahi A.; Arunan E. Why are Hydrogen Bonds Directional?. Chem. Sci. J. 2016, 128, 1571–1577. 10.1007/s12039-016-1156-3.

[ref45] Nulek T.; Klaysri R.; Cedeno R.; Nalaoh P.; Bureekaew S.; Promarak V.; Flood A. E. Separation of Etiracetam Enantiomers Using Enantiospecific Cocrystallization with 2-Chloromandelic Acid. ACS Omega 2022, 7, 19465–19473. 10.1021/acsomega.2c01165. 35721919 PMC9202017

[ref46] Mičová J.; Steiner B.; Koóš M.; Langer V.; Gyepesová D. Characterisation and X-ray crystallography of products from the Bucherer–Bergs reaction of methyl 2,3-O-isopropylidene-α-d-lyxo-pentodialdo-1,4-furanoside. Carbohydr. Res. 2003, 338 (19), 1917–1924. 10.1016/S0008-6215(03)00307-0. 14499568

[ref47] Rychlewska U.; Warzajtis B. Packing modes of (*R*,*R*)-tartaric acid esters and amides. Acta Crystallogr. 2000, B56, 833–848. 10.1107/S0108768100004274. 11006560

[ref48] Habgood M. Analysis of Enantiospecific and Diastereomeric Cocrystal Systems by Crystal Structure Prediction. Cryst. Growth Des. 2013, 13 (10), 4549–4558. 10.1021/cg401040p.

