# Supplementary material for: Exploring the Crystal Landscape of Mandelamide and Chiral Resolution via Cocrystallization
Source: Cryst Growth Des. 2024 Dec 10;25(1):1–12. doi: 10.1021/acs.cgd.3c01513 (PMC11697332; doi:10.1021/acs.cgd.3c01513)
Supplement: Supplementary file 1 — cg3c01513_si_001.pdf [file cg3c01513_si_001.pdf]

Supporting Information

Exploring the Crystal Landscape of Mandelamide and Chiral Resolution via Cocrystallization

Shan Huang, Deirbhile Fitzgerald, Samuel A. Koledoye, Stuart G. Collins, Anita R. Maguire, and Simon E. Lawrence

Contents

Synthesis of *S*-MDM	2
Synthesis of (±)-MDM	3
^{1}H and ^{13}C NMR spectra	4
FT-IR spectra	5
DSC plots	7
PXRD patterns	9
Ellipsoid plots	12
Hydrogen bonding in MDM (94 *S* : 6 *R*) (major component)	14
Hydrogen bonding in MDM (94 *S* : 6 *R*) (minor component)	16
2D fingerprint plots of *S*-MDM	17
Chiral HPLC data	18
Distinctive Bands (cm^{-1}) in the FTIR Spectra	19
Hydrogen bond geometries	20
Summary of the various contact contributions in *S*-MDM cocrystals	24
Slurry experiments and investigation of chiral resolution through cocrystallization	25
Proof of concept of resolution through cocrystallization	26
Chiral HPLC analysis of the solid S-MDM-L-Pro (1:2) recovered from 1:5 experiment	26
Summary of the reported cocrystals of mandelic acid and chiral coformers	27
References	34

(*S*)-2-Hydroxy-2-phenylacetamide[1]

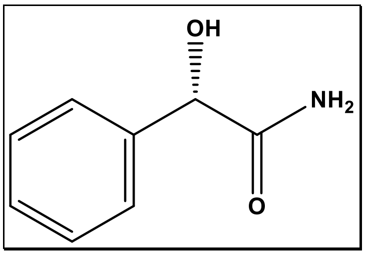

This was synthesized following a literature procedure.[1] (*S*)-2-Hydroxy-2-phenylacetic acid (6.00 g, 39 mmol) was dissolved in MeOH (60 mL) and cooled to 0°C. Acetyl chloride (3.38 mL, 47 mmol) was added dropwise to the solution. The reaction mixture was allowed to warm to room temperature and stirred for 24 h. The resulting solution was concentrated under reduced pressure to give a colourless liquid, which was dissolved in MeOH (25 mL). Aqueous NH_3 (85 mL, 35% w/v) was added, and the solution was stored at below 5°C for a further 24 h. The solution was then concentrated under reduced pressure to give a white solid *S*-MDM I, which was recrystallised from hot ethanol to yield white plates (2.45 g, 41%). mp 117-120°C, (lit.[2] 118–120 °C); $[\alpha]^{25}_{D}$= +51.5 (c 1.05 in EtOH) [Lit.[3], $[\alpha]^{25}_{D}$= +57 (c 1.05 in EtOH)]; ν_{max}/cm^{-1} (ATR): 3346, 3182, 1680, 1655, 1586, 1452, 1292, 1098, 1055. ^{1}H NMR (400 MHz, CD_3OD): 4.99 (1H, s, C*H*OH), 7.22-7.37 (3H, m, aromatic *H*), 7.46 (2H, d, *J* = 9.5 Hz, aromatic *H*); ^{13}C NMR (100.6 MHz, CD_3OD): 75.4 (*C*HOH), 128.0 (aromatic *C*H), 129.2 (aromatic *C*H), 129.4 (aromatic *C*H), 141.7 (aromatic C_q), 178.6 (*C*=O).

(±)-2-Hydroxy-2-phenylacetamide[1]

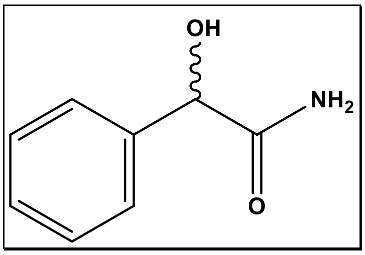

This was synthesized following a literature procedure.[1] (±)-Hydroxy-2-phenylacetic acid (8.09 g, 53 mmol) was dissolved in MeOH (70 mL) and cooled to 0°C. Acetyl chloride (9.95 mL, 139 mmol) was added dropwise to the solution. The reaction mixture was allowed to warm to room temperature and stirred for 24 h. The resulting solution was concentrated under reduced pressure to give a colourless liquid, which was dissolved in MeOH (35 mL). Aqueous NH_3 (100 mL, 35% w/v) was added, and the solution was stored at below 5°C for a further 24 h. The solution was then concentrated under reduced pressure to give a white solid (±)-MDM I, which was recrystallised from hot ethanol to yield white plates (6.92 g, 86%). mp by DSC 133-135° C(lit.[4] 132-135 °C); ν_{max}/cm^{-1} (ATR): 3380, 3251, 1650, 1635, 1597, 1444, 1056; ^{1}H NMR (400 MHz, CD_3OD): 4.99 (1H, s, C*H*OH), 7.19-7.37 (3H, m, aromatic *H*), 7.45 (2H, d, J = 8.5 Hz, aromatic *H*); ^{13}C NMR (100.6 MHz, CD_3OD): 75.4 (*C*HOH), 128.0 (aromatic *C*H), 129.1 (aromatic *C*H), 129.4 (aromatic *C*H), 141.7 (aromatic C_q), 178.6 (*C*=O).

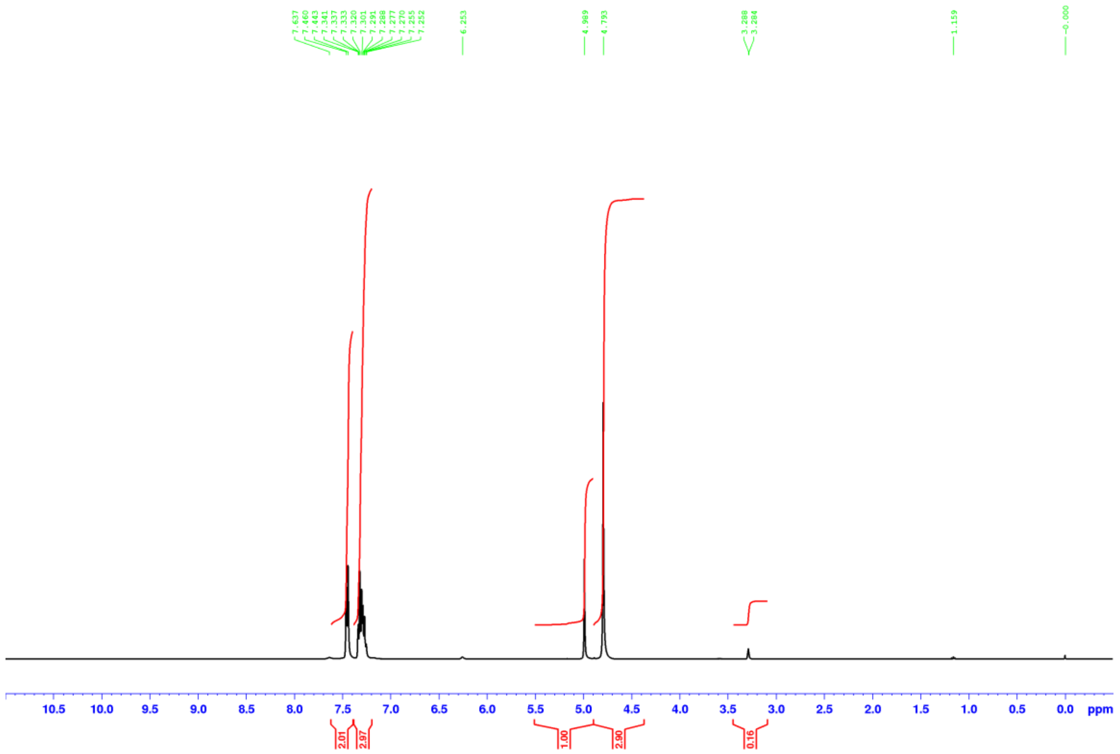

Figure S1. ^{1}H NMR spectrum of *S*-MDM (CD_3OD, 400 MHz). Signals at 3.29 and 4.79 ppm due to MeOH and total exchangeable hydrogens.

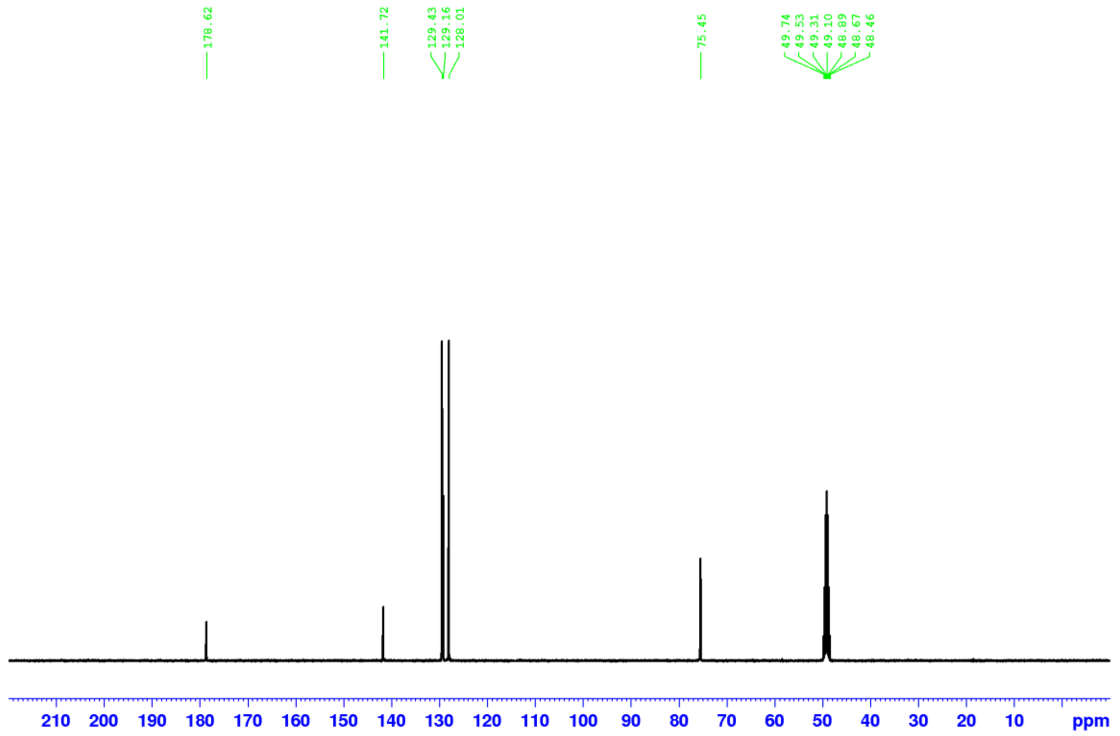

Figure S2. ^{13}C NMR spectrum of *S*-MDM (CD_3OD, 100.6 MHz). Signals at 48.4-49.7 ppm due to CD_3OD.

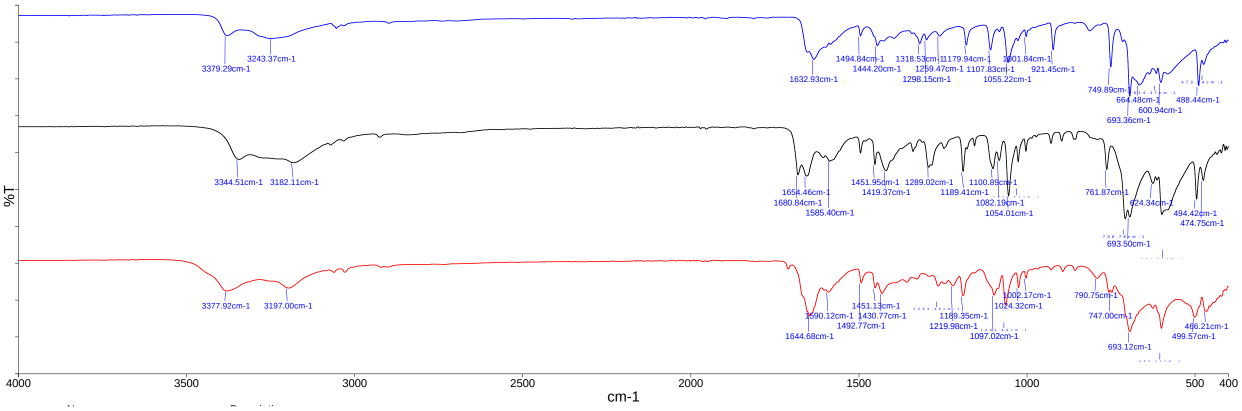

Figure S3. IR spectra of (±)-MDM (blue), *S*-MDM (black) and MDM (94 *S* : 6 *R*) (red).

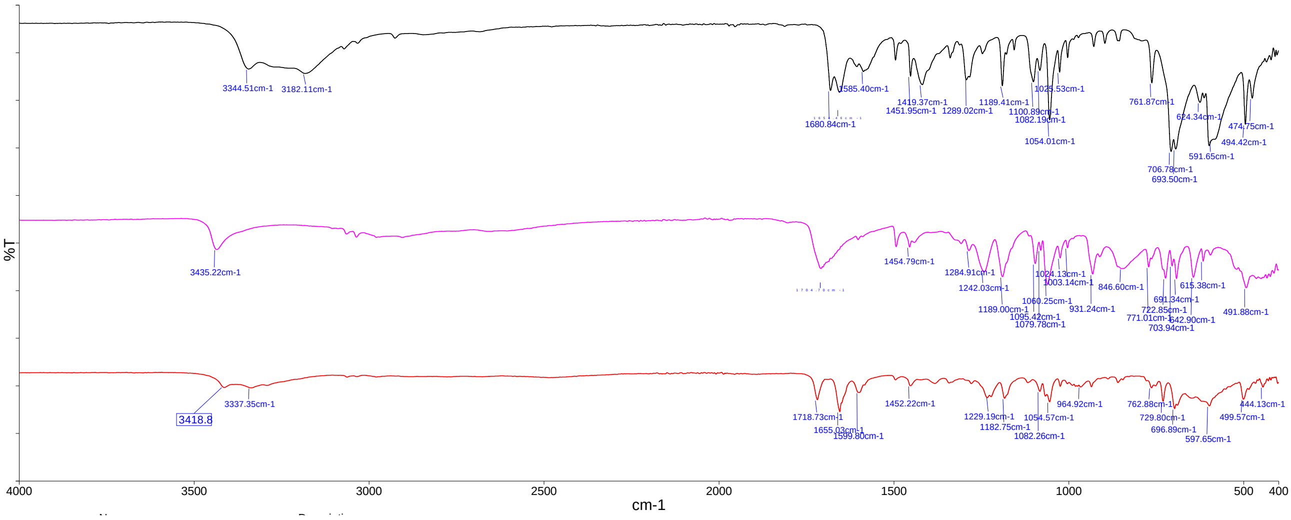

Figure S4. IR spectra of *S*-MDM (black), *S*-MDA (pink) and *S*-MDM-*S*-MDA cocrystal (red).

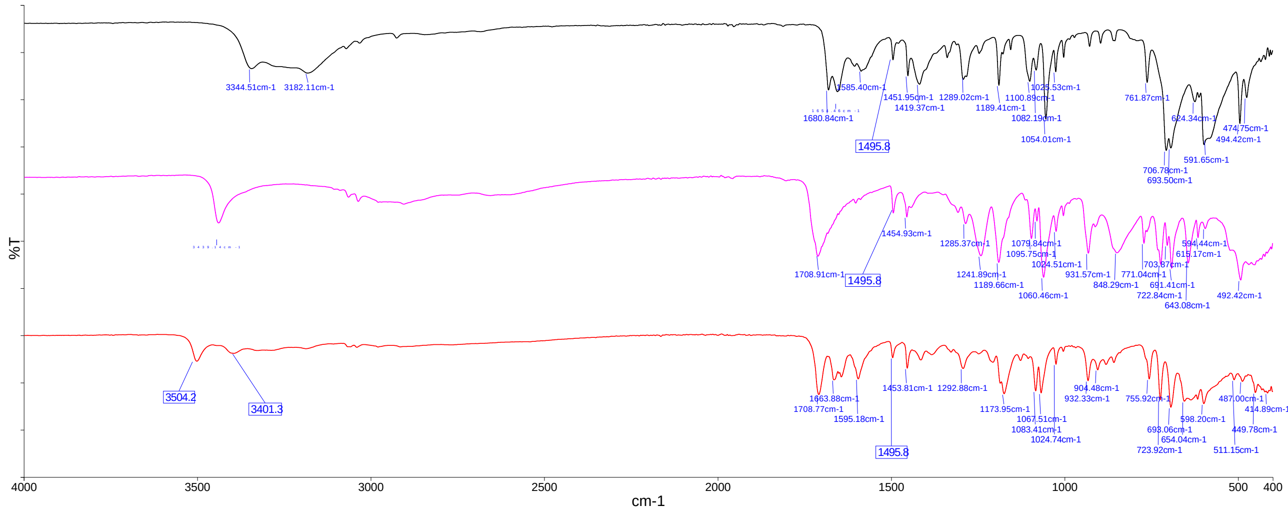

Figure S5. IR spectra of *S*-MDM (black), *R*-MDA (pink) and *S*-MDM-*R*-MDA cocrystal (red).

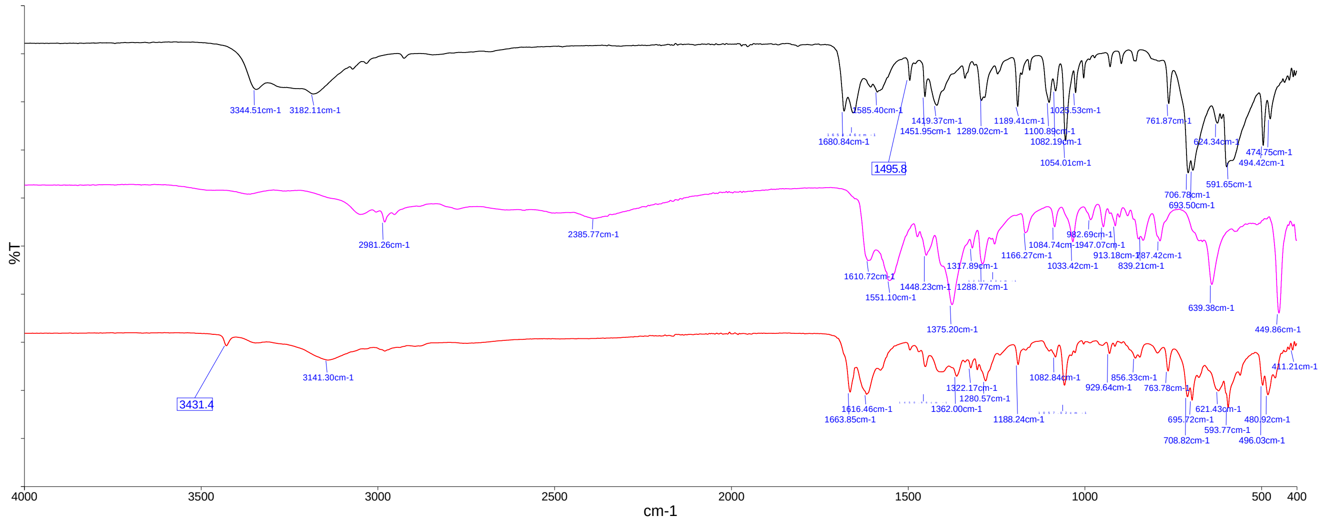

Figure S6. IR spectra of *S*-MDM (black), L-Pro (pink) and *S*-MDM-L-Pro cocrystal (red).

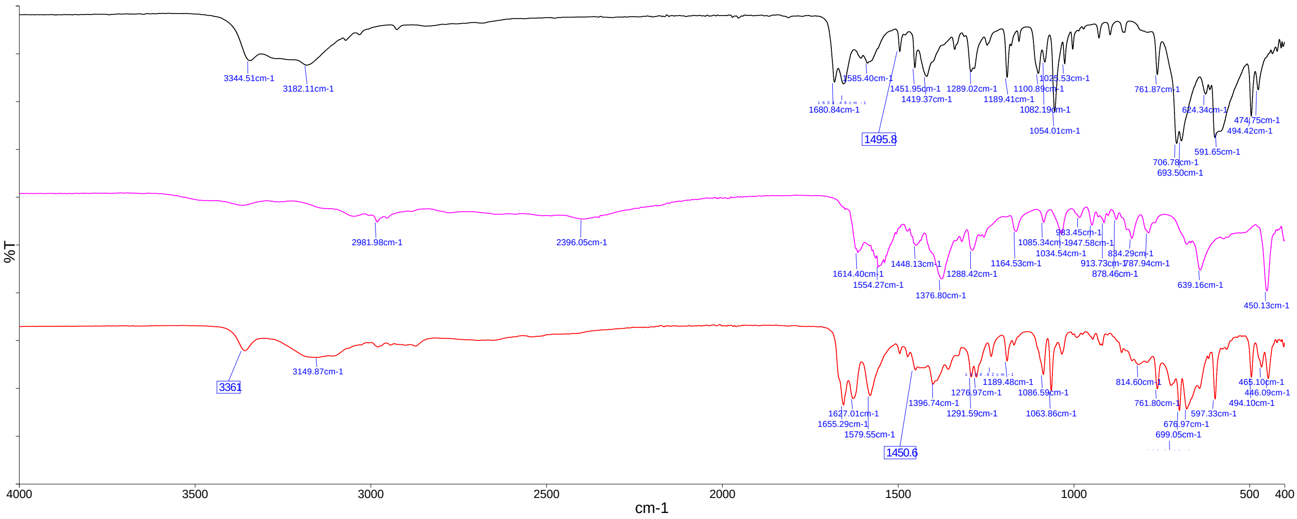

Figure S7. IR spectra of *S*-MDM (black), D-Pro (pink) and *S*-MDM-D-Pro cocrystal (red).

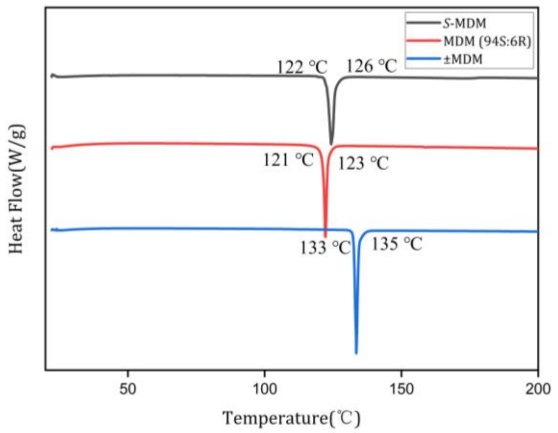

Figure S8. DSC curves of (±)-MDM, *S*-MDM and MDM (94 *S* : 6 *R*).

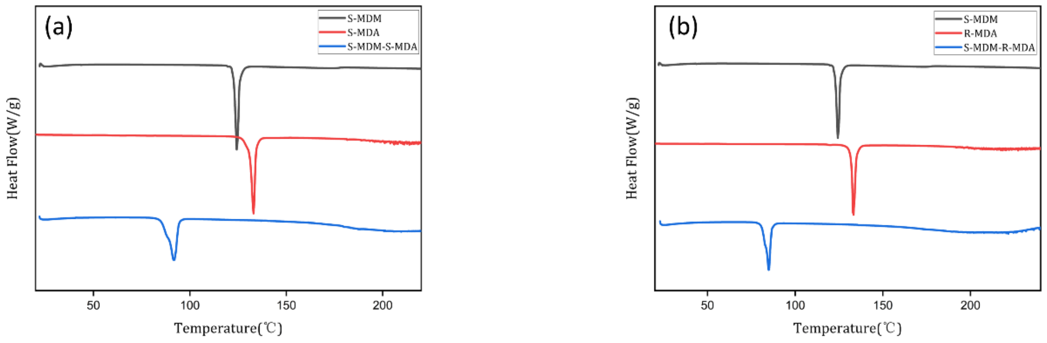

Figure S9. DSC plots of *S*-MDM-*R*/*S*-MDA cocrystal pair.

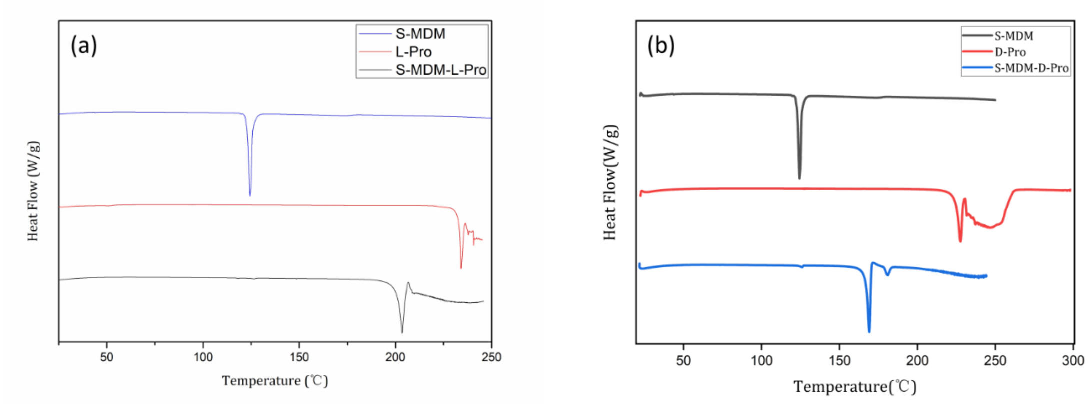

Figure S10. DSC plots of S-MDM-L/D-Pro cocrystal pair.

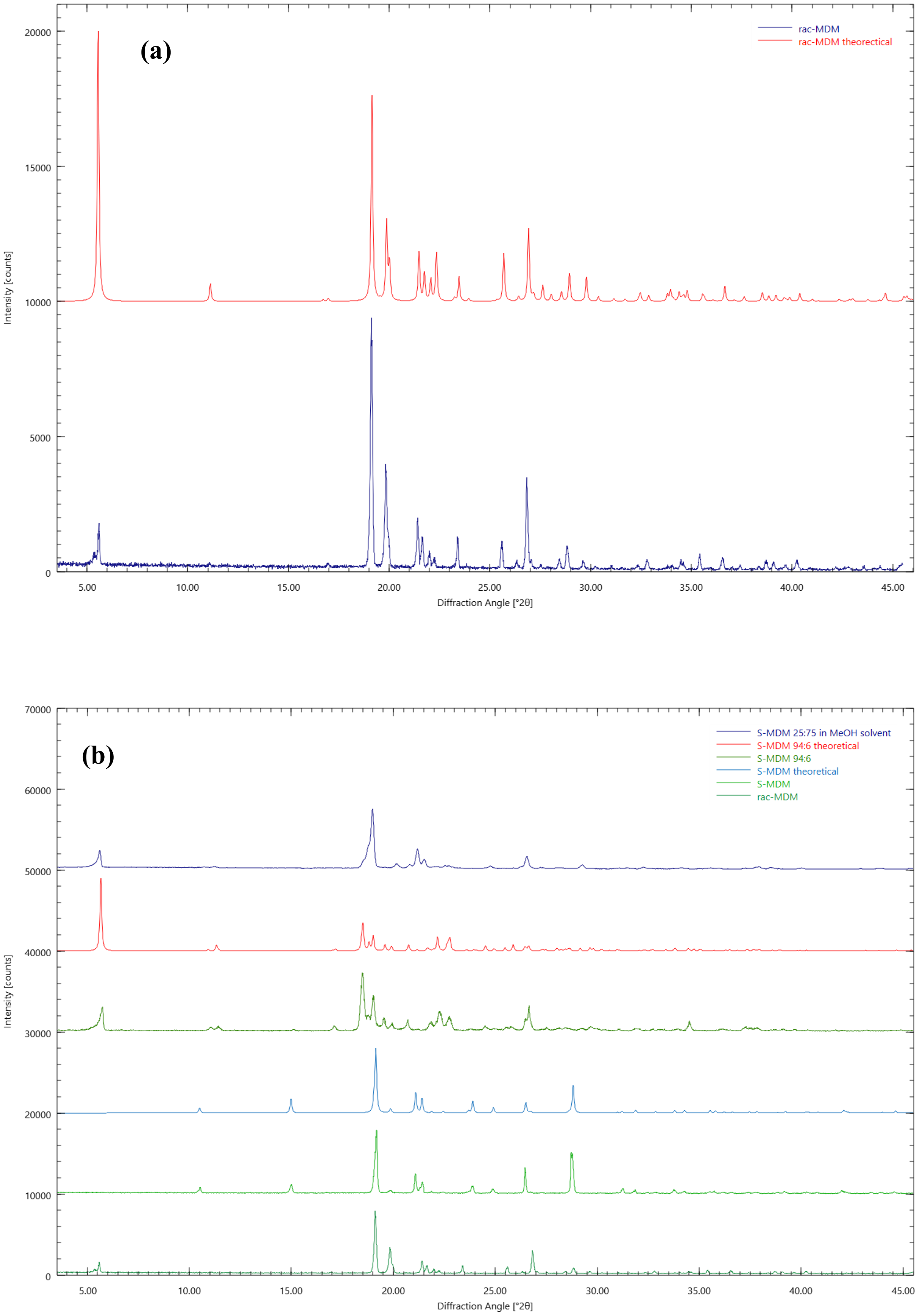

Figure S11. PXRD patterns of (a) (±)-MDM and (b) *S*-MDM, MDM (94 *S* : 6 *R*), (±)-MDM and an equimolar sample of (±)-MDM and *R*-MDM (25% S, 75% R) after dissolution and crystallization from methanol.

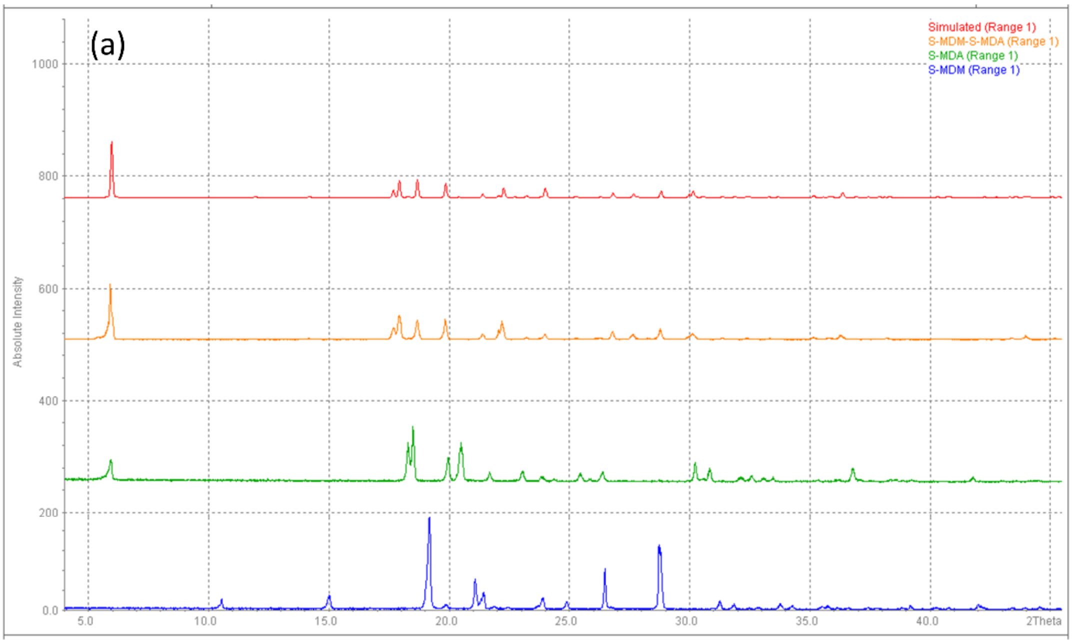

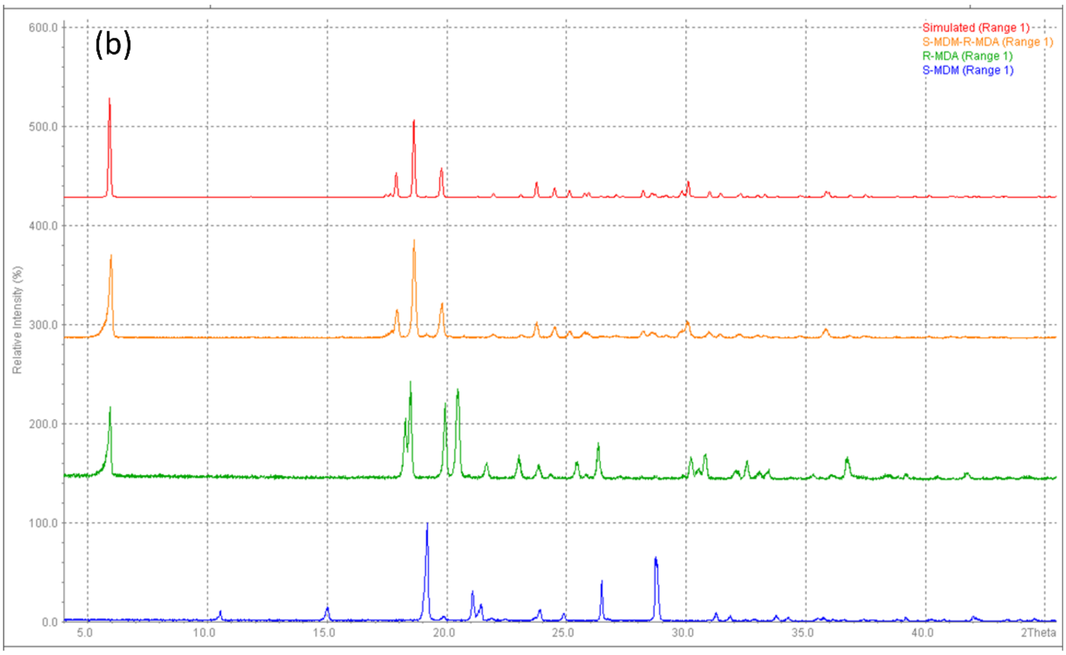

Figure S12. PXRD pattern of *S*-MDM-*R*/*S*-MDA cocrystal pair.

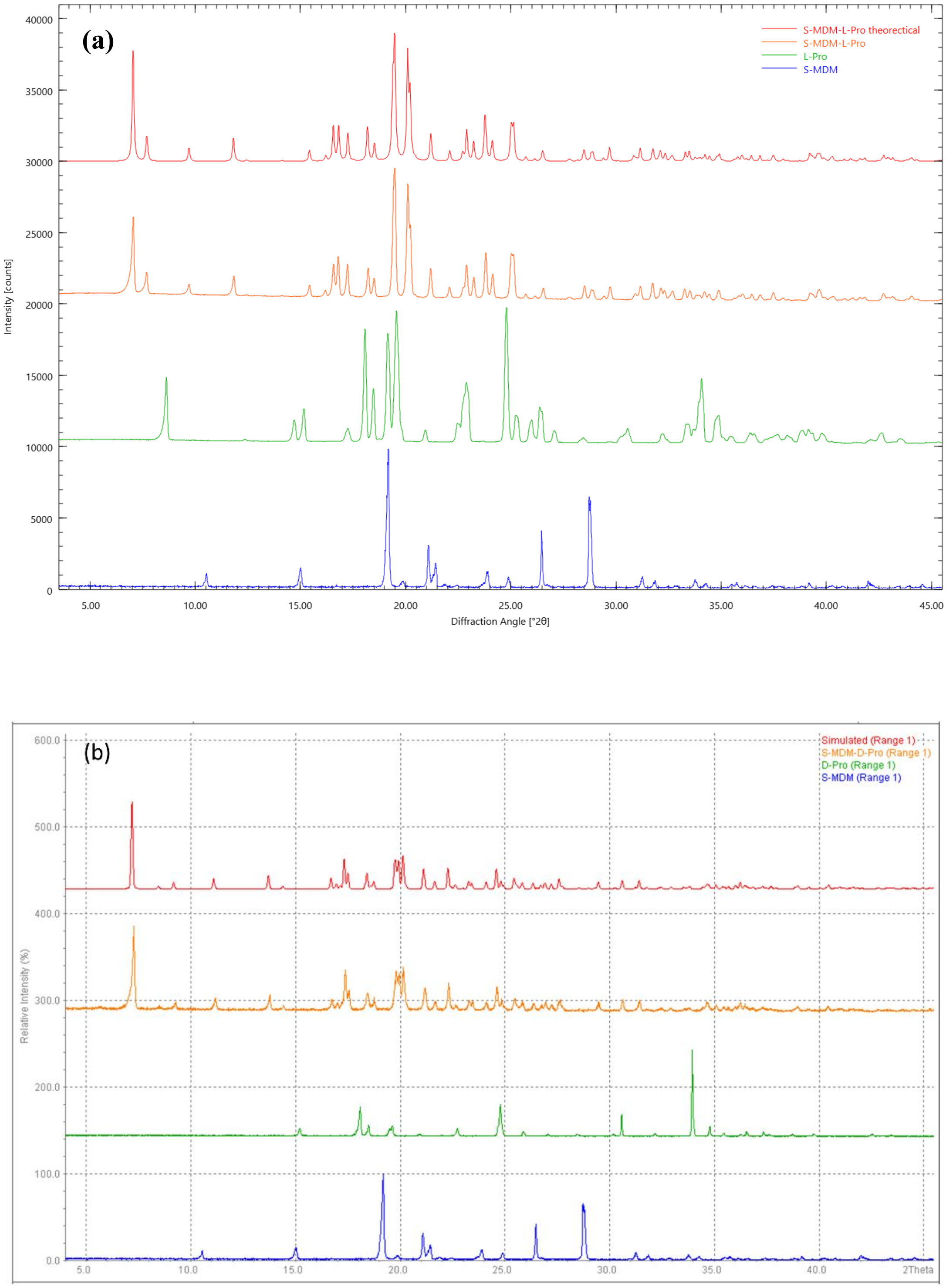

Figure S13. PXRD pattern of S-MDM-L/D-Pro cocrystal pair.

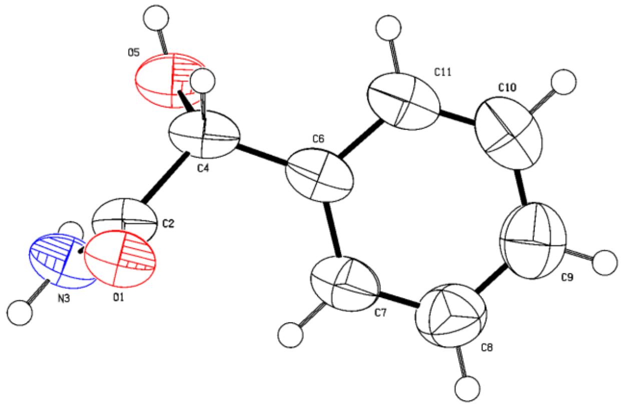

Figure S14. Ellipsoid plot of (±)-MDM.

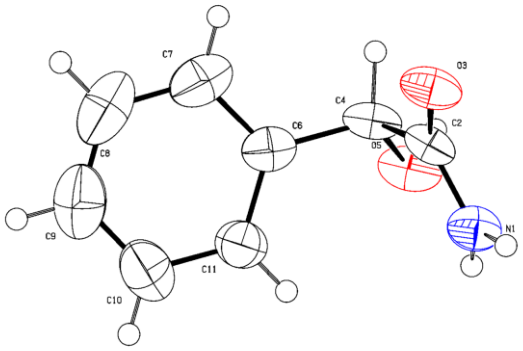

Figure S15. Ellipsoid plot of *S*-MDM.

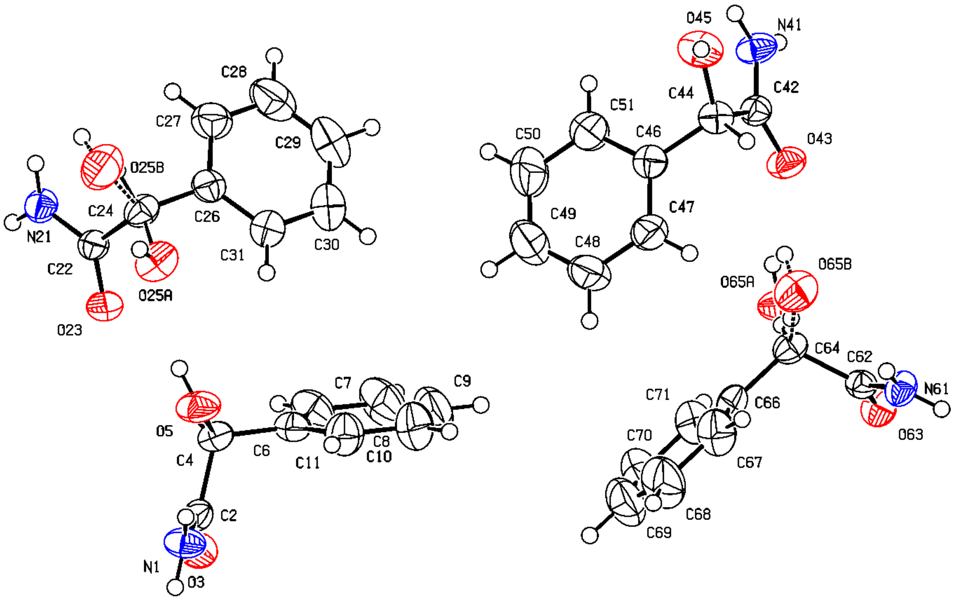

Figure S16. Ellipsoid plot of MDM (94 *S* : 6 *R*).

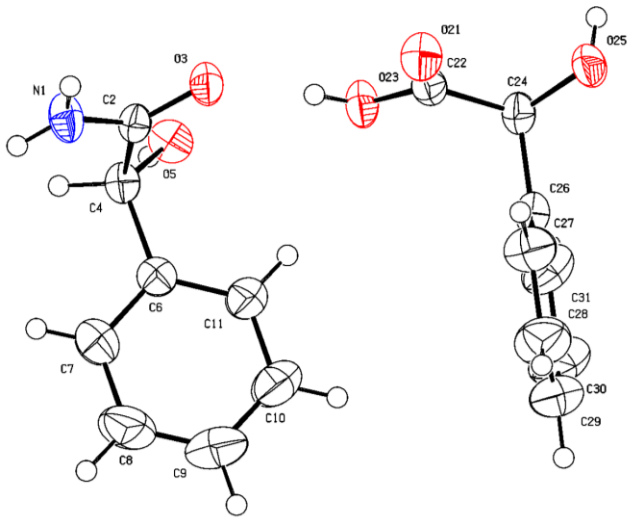

Figure S17. Ellipsoid plot *S*-MDM-*S*-MDA.

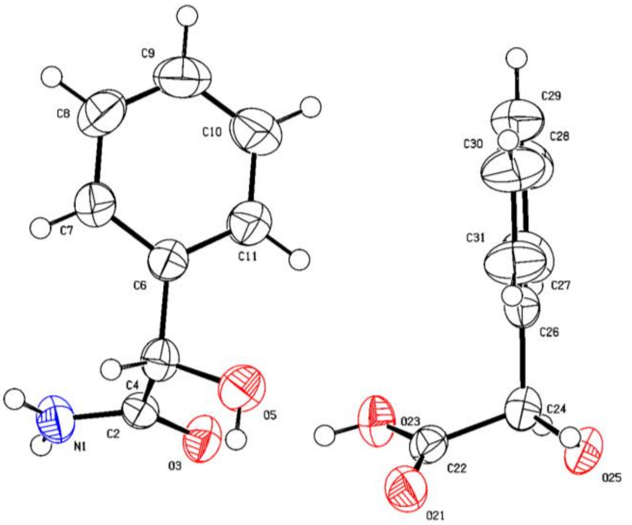

Figure S18. Ellipsoid plot of *S*-MDM-*R*-MDA.

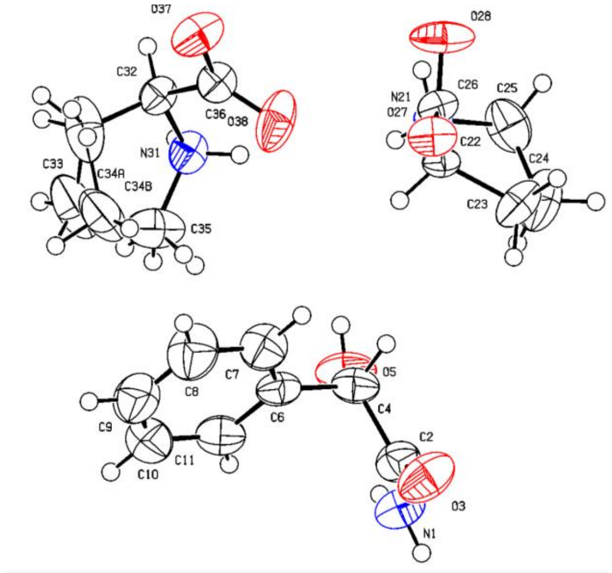

Figure S19. Ellipsoid plot *S*-MDM-L-Pro.

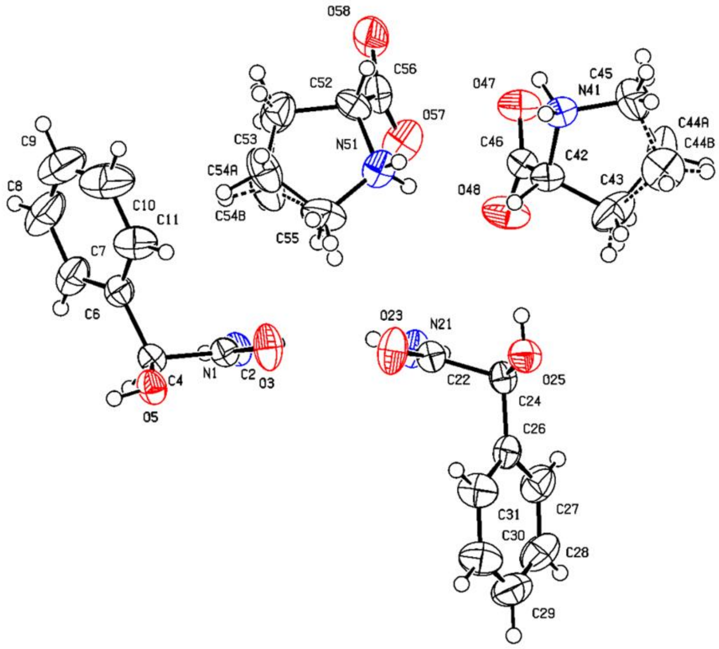

Figure S20. Ellipsoid plot of *S*-MDM-D-Pro.

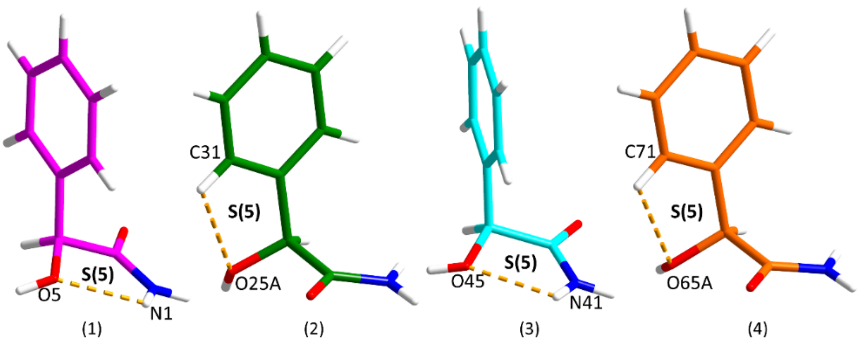

(a)

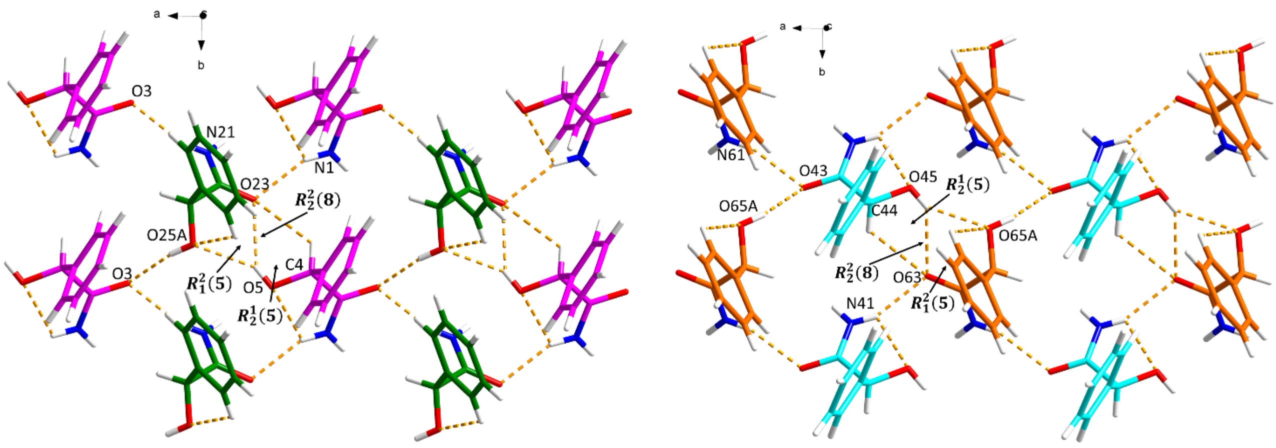

(b)

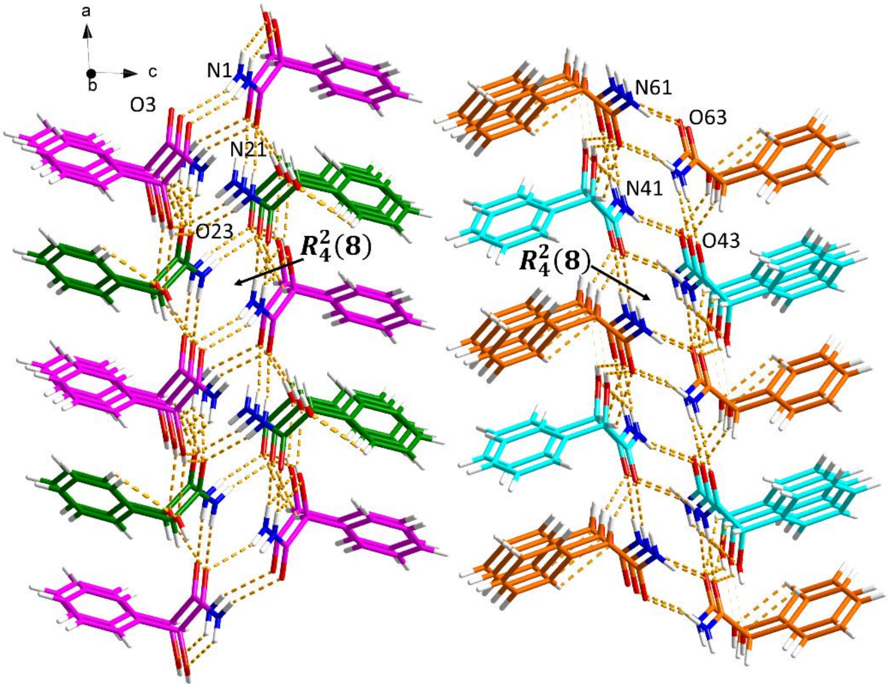

(c)

Figure S21. Hydrogen bonding in MDM (94 *S* : 6 *R*) (major component of the disordered structure): (a) intramolecular interactions in the four *S*-MDM molecules, (b) 2D hydrogen-bonding network along the *c* axis, and (c) 3D hydrogen-bonding network along the *b* axis.

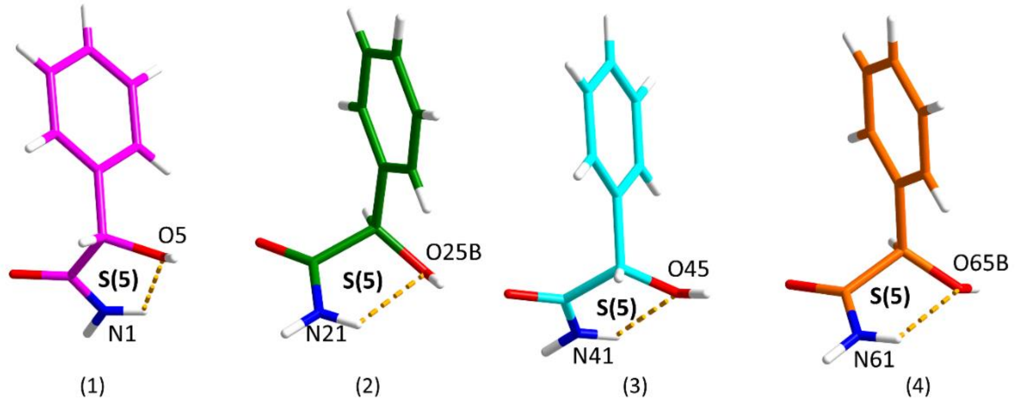
O5
S(5)
N1
(1)
O25B
S(5)
N21
(2)
O45
S(5)
N41
(3)
O65B
S(5)
N61
(4)

(a)

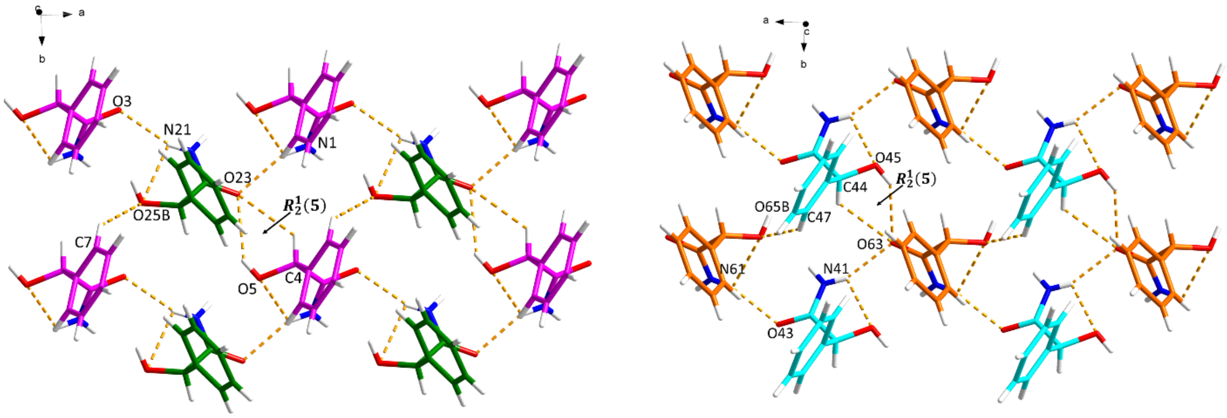
a
b
c
O3
N21
N1
O23
$R_2^1(5)$
O25B
C7
O5
C4
a
c
b
O45
C44
$R_2^1(5)$
O65B
C47
O63
N61
N41
O43

(b)

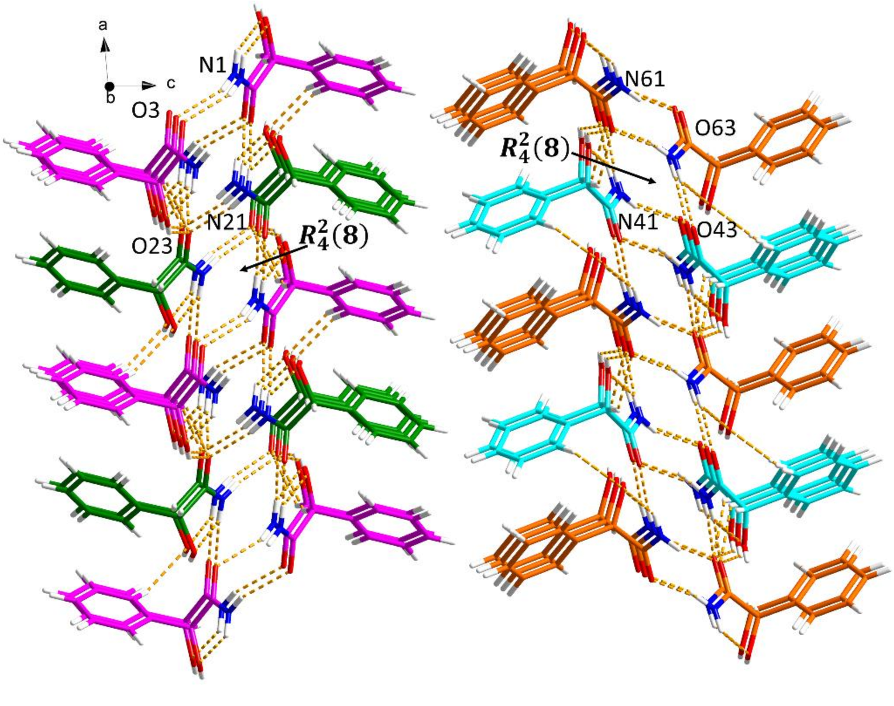
a
c
b
N1
O3
N21
O23
$R_4^2(8)$
N61
O63
$R_4^2(8)$
N41
O43

(c)

Figure S22. Hydrogen bonding in MDM (94 *S* : 6 *R*) (minor component of the disordered structure): (a) intramolecular interactions in the four MDM molecules (molecules 1 and 3 are *S*-MDM, while molecules 2 and 4 are *R*-MDM), (b) 2D hydrogen-bonding network along the *c* axis, and (c) 3D hydrogen-bonding network along the *b* axis.

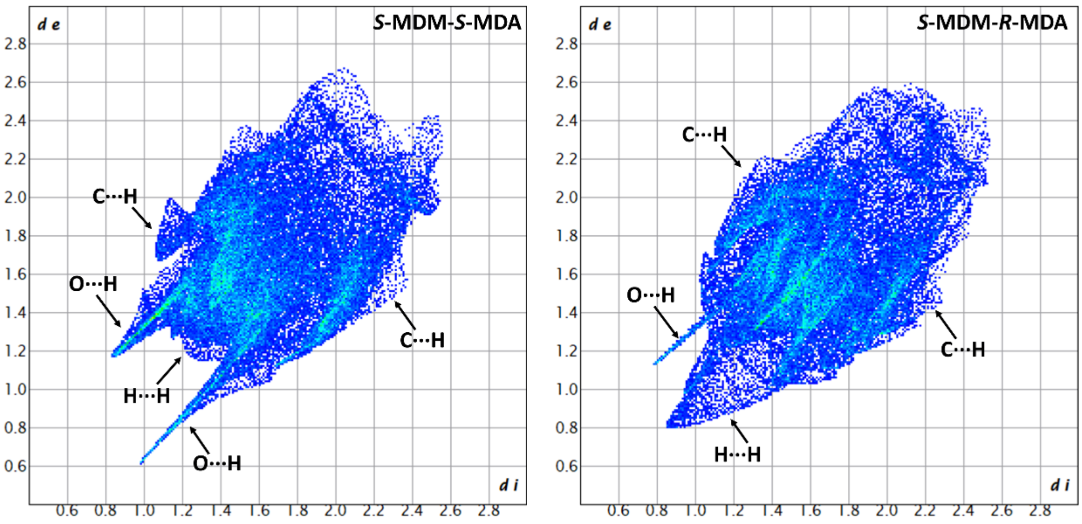

(a)

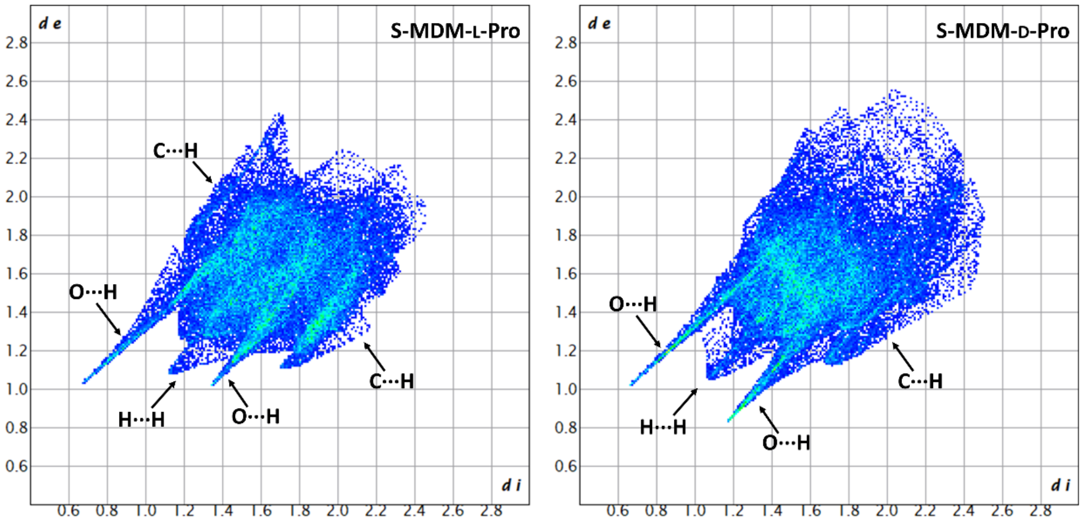

(b)

Figure S23. 2D fingerprint plots of *S*-MDM in (a) *S*-MDM-*S*/*R*-MDA cocrystal pair and (b) *S*-MDM-L/D-Pro cocrystal pair.

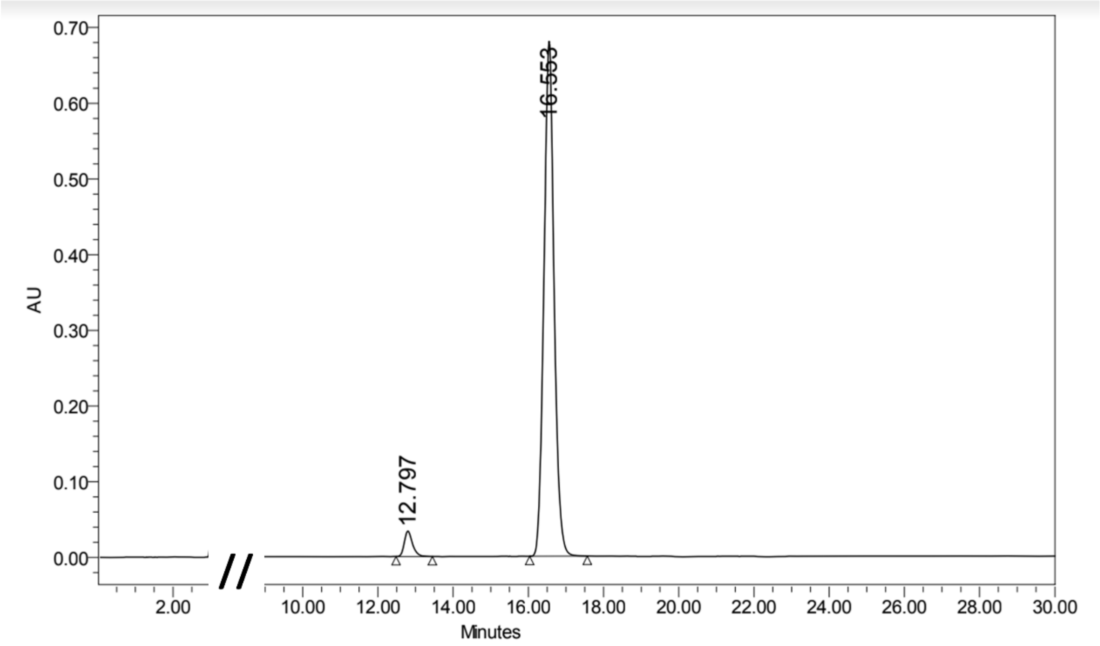

	RT	Area	% Area	Height
1	12.797	505860	3.80	33408
2	16.553	12793348	96.20	680250

Figure S24. Chiral HPLC data of commercial *S*-MDM from Sigma Aldrich.

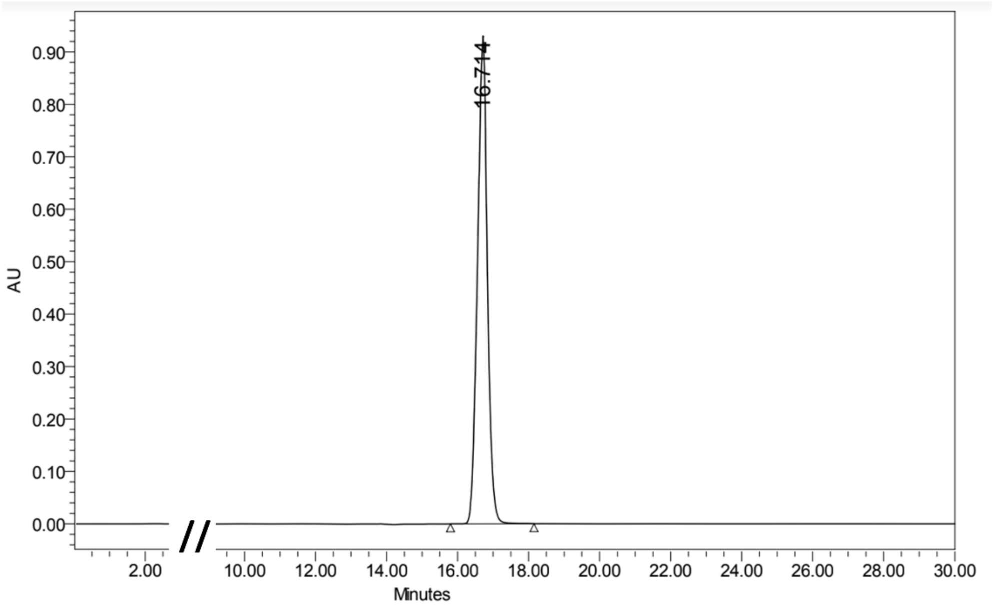

	RT	Area	% Area	Height
1	16.714	17904751	100.00	929904

Figure S25. Chiral HPLC data of synthesized *S*-MDM.

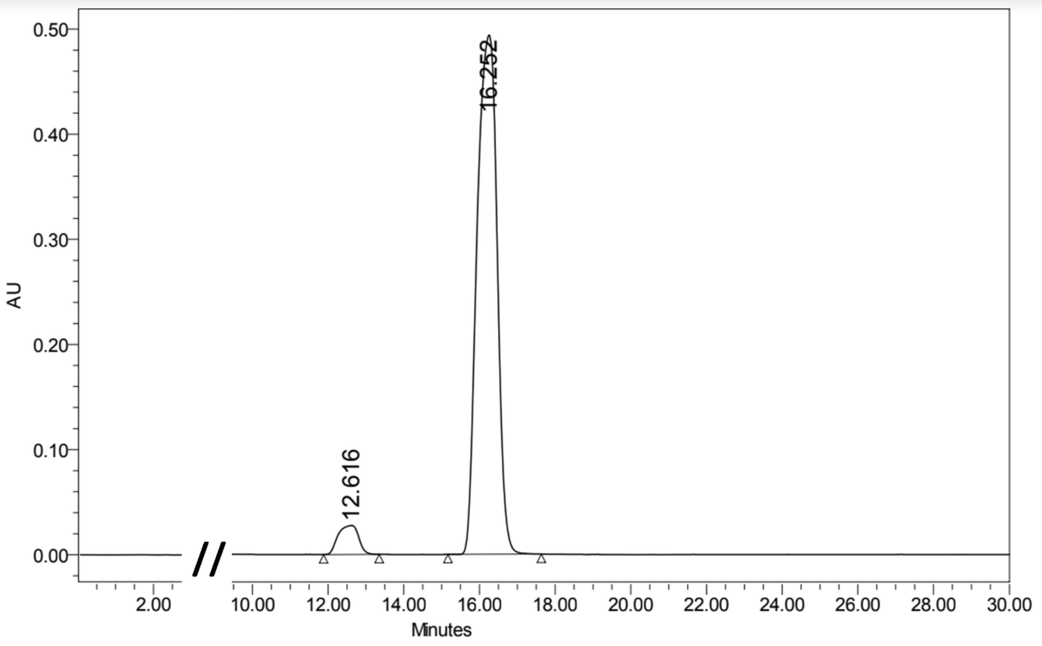

	RT	Area	% Area	Height
1	12.616	1033483	5.40	27552
2	16.252	18117931	94.60	493644

Figure S26. Chiral HPLC data of single crystal of MDM (94 *S* : 6 *R*).

Table S1. Distinctive Bands (cm^{-1}) in the FTIR Spectra of MDM and Cocrystals.

solid form	$\nu_{\mathrm{NH_2}}$	ν_{OH}	$\nu_{C=O}$
(±)-MDM	3379	3243	1633
S-MDM	3345	3182	1681
MDM (94 *S* : 6 *R*)	3378	3197	1644
S-MDM-S-MDA	3419	3337	1719
S-MDM-R-MDA	3504	3401	1709
S-MDM-L-Pro	3431	3141	1664
S-MDM-D-Pro	3361	3150	1655

Table S2. Hydrogen bonds (Å, °) in (±)-MDM[a]

		D-H···A	D-H	H···A	D···A	D-H···A	ARU (J)
1		N3-H3A···O1	0.86	2.15	2.984(3)	164	[2646.01]
2	Intra	N3-H3B···O5	0.86	2.29	2.658(3)	106	
3		N3-H3B···O1	0.86	2.21	3.013(3)	156	[4554.01]
4		O5-H5···O1	0.82	1.97	2.772(3)	166	[4564.01]
5		C7-H7···O5	0.93	2.52	3.433(4)	166	[4555.01]

[a] Symmetry codes: [4555.] = [4_566] = x, 1/2-y ,1/2+z; [4564.] = [4_575] = x, 3/2-y, -1/2+z; [4554.] = [4_565] = x, 1/2-y, -1/2+z; [2646.] = [2_646] = 1-x, -1/2+y, 3/2-z.

Table S3. Hydrogen bonds (Å, °) in *S*-MDM[a]

		D-H···A	D-H	H···A	D···A	D-H···A	ARU (J)
1		N1-H1A···O5	0.86	2.09	2.894(2)	155	[4656.01]
2	Intra	N1-H1B···O5	0.86	2.36	2.709(2)	105	
3		N1-H1B···O3	0.86	2.12	2.920(2)	155	[4646.01]
4		O5-H5···O3	0.82	1.89	2.7052(17)	171	[4746.01]

[a] Symmetry codes: [4656.] = [3_656] = 1-x, 1/2+y, 3/2-z; [4746.] = [3_746] = 2-x, -1/2+y, 3/2-z; [4646.] = [3_646] = 1-x, -1/2+y, 3/2-z.

Table S4. Hydrogen bonds (Å, °) in MDM (94 *S* : 6 *R*)[a]

		D-H···A	D-H	H···A	D···A	D-H···A	ARU (J)
1		N1-H1A···O3	0.86	2.22	3.039(2)	159	[2556.03]
2	Intra	N1-H1B···O5	0.86	2.2	2.592(2)	107	
3		N1-H1B···O23	0.86	2.16	2.969(2)	156	[1565.01]
4		O5-H5···O23	0.82	2.41	3.098(3)	142	[1555.01]
5		O5-H5···O25A	0.82	2.23	2.950(2)	146	[1555.01]
6		N21-H21A···O23	0.86	2.1	2.944(3)	169	[2646.01]
7		N21-H21B···O3	0.86	2.18	3.007(2)	162	[1645.03]
8	Intra	N21-H21B···O25B	0.86	2.41	2.717(18)	102	
9		O25-H25A···O3	0.82	1.95	2.762(2)	169	[1655.03]
10		N41-H41A···O43	0.86	2.24	3.049(2)	157	[2647.04]
11	Intra	N41-H41B···O45	0.86	2.2	2.585(2)	107	
12		N41-H41B···O63	0.86	2.14	2.950(2)	156	[1645.02]
13		O45-H45···O63	0.82	2.42	3.063(3)	136	[1655.02]
14		O45-H45···O65A	0.82	2.19	2.923(2)	149	[1655.02]
15		N61-H61A···O63	0.86	2.13	2.970(3)	167	[2557.02]
16		N61-H61B···O43	0.86	2.16	2.995(2)	162	[1565.04]
17	Intra	N61-H61B···O65B	0.86	2.39	2.700(14)	102	
18		O65A-H65A···O43	0.82	2	2.743(2)	151	[1555.04]
19		C4-H4···O23	0.98	2.58	3.271(3)	128	[1555.01]
20		C7-H7···O25B	0.93	2.28	3.021(18)	136	[1455.01]
21	Intra	C31-H31···O25A	0.93	2.43	2.753(3)	100	
22		C44-H44···O63	0.98	2.55	3.237(3)	127	[1655.02]
23		C47-H47···O65B	0.93	2.28	3.035(14)	138	[1555.02]
24	Intra	C71-H71···O65A	0.93	2.43	2.752(3)	100	

[a] Symmetry codes: [1655.] = [1_655] = 1+x, y, z; [1645.] = [1_645] = 1+x, -1+y, z; [2646.] = [2_646] = 1-x, -1/2+y, 1-z; [1565.] = [1_565] = x, 1+y, z; [2556.] = [2_556] = -x, 1/2+y, 1-z; [2647.] = [2_647] = 1-x, -1/2+y, 2-z; [2557.] = [2_557] = -x, 1/2+y, 2-z; [1455.] = [1_455] = -1+x, y, z.

Table S5. Hydrogen bonds (Å, °) in the *S*-MDM-*S*-MDA cocrystal [a]

	D-H···A	D-H	H···A	D···A	D-H···A	ARU (J)
1	N1-H1A···O23	0.86	2.24	3.077(2)	164	[1655.02]
2	N1-H1B···O25	0.86	2.16	2.859(2)	139	[1645.02]
3	O5-H1···O21	0.82	2.17	2.897(2)	148	[1545.02]
4	O5-H1···O25	0.82	2.44	3.109(2)	139	[1545.02]
5	O23-H23···O3	0.82	1.78	2.5946(19)	174	[1555.01]
6	O25-H25···O5	0.82	2.08	2.873(2)	164	[3466.01]
7	C4-H4···O25	0.98	2.56	3.387(3)	142	[1645.02]
8	C11-H11···O23	0.93	2.54	3.370(3)	149	[1555.02]
9	C31-H31···O3	0.93	2.52	3.389(3)	156	[1455.01]

[a] Symmetry codes: [1545.] = [1_545] = x, -1+y,z; [1455.] = [1_455] = -1+x, y, z; [1655.] = [1_655] = 1+x, y, z; [1645.] = [1_645] = 1+x,-1+y,z; [3466.] = [4_466] = -1/2+x,3/2-y,1-z.

Table S6. Hydrogen bonds (Å, °) in the *S*-MDM-*R*-MDA cocrystal [a]

	D-H···A	D-H	H···A	D···A	D-H···A	ARU (J)
1	N1-H1A···O21	0.86	2.37	3.206(3)	165	[1545.02]
2	N1-H1B···O25	0.86	2.08	2.938(3)	177	[1645.02]
3	O5-H5···O21	0.82	2.26	2.947(3)	142	[3566.02]
4	O5-H5···O25	0.82	2.59	3.323(3)	151	[3566.02]
5	O23-H23···O3	0.82	1.88	2.626(2)	150	[1555.01]
6	O23-H23···O5	0.82	2.55	3.078(3)	124	[1555.01]
7	O25-H25···O3	0.82	2.06	2.846(2)	162	[1565.01]

[a] Symmetry codes: [1565.] = [1_565] = x, 1+y, z; [3566.] = [4_566] = 1/2+x, 3/2-y, 1-z; [1545.] = [1_545] = x, -1+y, z; [1645.] = [1_645] = 1+x, -1+y, z.

Table S7. Hydrogen bonds (Å, °) in the *S*-MDM-L-Pro cocrystal [a]

		D-H···A	D-H	H···A	D···A	D-H···A	ARU (J)
1		N1-H1A···O37	0.86	2.05	2.909(3)	174	[2564.02]
2	Intra	N1-H1B···O5	0.86	2.21	2.604(4)	107	
3		O5-H5···O27	0.82	1.87	2.688(3)	172	[1455.03]
4		N21-H21A···O27	0.89	1.98	2.823(3)	157	[1455.03]
5		N21-H21A···O38	0.89	2.58	2.905(4)	103	[1555.02]
6	Intra	N21-H21B···O28	0.89	2.18	2.654(3)	113	
7		N21-H21B···O28	0.89	2.07	2.807(3)	140	[3466.03]
8		N31-H31A···O37	0.89	1.87	2.715(3)	157	[1455.02]
9		N31-H31A···O38	0.89	2.58	3.263(3)	134	[1455.02]
10		N31-H31B···O27	0.89	2.2	2.981(3)	146	[1455.03]
11	Intra	N31-H31B···O38	0.89	2.21	2.637(3)	109	
12		C22-H22···O38	0.98	2.57	2.962(4)	104	[1555.02]
13		C32-H32···O3	0.98	2.46	3.360(4)	152	[2565.01]

[a] Symmetry codes: [1455.] = [1_455] = -1+x, y, z; [2564.] = [2_564] = 1/2-x, 1-y, -1/2+z; [3466.] = [4_466] = -1/2+x, 3/2-y, 1-z; [2565.] = [2_565] = 1/2-x, 1-y, 1/2+z.

Table S8. Hydrogen bonds (Å, °) in the *S*-MDM-D-Pro cocrystal [a]

		D-H···A	D-H	H···A	D···A	D-H···A	ARU (J)
1		N1-H1A···O23	0.86	2.07	2.914(3)	166	[1455.02]
2		N1-H1B···O5	0.86	2.16	3.011(3)	169	[1455.01]
3		O5-H5···O58	0.82	1.83	2.630(3)	164	[1554.04]
4		N21-H21A···O3	0.86	2.14	2.963(3)	160	[1555.01]
5		N21-H21B···O25	0.86	2.25	3.099(3)	168	[1455.02]
6		O25-H25···O48	0.82	1.87	2.670(3)	164	[1655.03]
7		N41-H41A···O5	0.89	2.23	3.048(3)	153	[1556.01]
8	Intra	N41-H41A···O47	0.89	2.16	2.622(3)	111	
9		N41-H41B···O47	0.89	1.88	2.760(3)	168	[1655.03]
10		N51-H51A···O48	0.89	2.39	3.115(3)	139	[1655.03]
11	Intra	N51-H51A···O57	0.89	2.19	2.650(3)	112	
12		N51-H51B···O57	0.89	1.89	2.759(3)	164	[1655.04]
13		C28-H28···O58	0.93	2.53	3.220(4)	132	[2656.04]

[a] Symmetry codes: [1554.] = [1_554] = x, y, -1+z; [1455.] = [1_455] = -1+x, y, z; [1655.] = [1_655] = 1+x, y, z; [2656.] = [2_656] = 1-x, 1/2+y, 1-z; [1556.] = [1_556] = x, y, 1+z.

Table S9. Summary of the various contact contributions in *S*-MDM cocrystals.

	H-H	O-H	C-H	C-O	N-H	N-O	O-O
***S*-MDM-*S*-MDA**	44.3%	27.3%	24.6%	1.2%	1.9%	0.2%	0.5%
***S*-MDM-*R*-MDA**	45.0%	22.0%	26.2%	0.3%	1.5%	0.3%	4.6%
***S*-MDM-L-Pro**	56.6%	25.7%	15.6%	0	2.1%	0	0
***S*-MDM-D-Pro**	53.4%	27.1%	17.0%	0.7%	1.6%	0.2%	0

Table S10. Slurry experiments and investigation of chiral resolution through cocrystallization.

**Starting Materials**	**Ratio**	**Product**	**Chiral HPLC % Area**			**PXRD Results**
			***R*-MDM**	***S*-MDM**	**%ee**	
(±)-MDM +L-Pro	1:1	Solid	39.11	60.89	21.8% (*S*)	*S*-MDM-L-Pro (1:2) + *R*-MDM-L-Pro (1:1) + (±)-MDM
		Mother liquor	54.21	45.79	8.4% (*R*)	
(±)-MDM +L-Pro	1:2	Solid	33.60	66.40	32.8% (*S*)	*S*-MDM-L-Pro (1:2) + *R*-MDM-L-Pro (1:1)
		Mother liquor	68.07	31.93	36.1% (*R*)	
(±)-MDM +L-Pro	1:3	Solid	17.64	82.36	64.7% (*S*)	*S*-MDM-L-Pro (1:2) + (small amount of) L-Pro or *R*-MDM-L-Pro (1:1)
		Mother liquor	60.03	39.97	20.1% (*R*)	
(±)-MDM +L-Pro	1:4	Solid	10.99	89.01	78.0% (*S*)	*S*-MDM-L-Pro (1:2) + L-Pro + (±)-MDM
		Mother liquor	63.29	36.71	25.7% (*R*)	
(±)-MDM +L-Pro	1:5	Solid	9.74	90.26	80.5% (*S*)	*S*-MDM-L-Pro (1:2) + L-Pro + (±)-MDM
		Mother liquor	65.98	34.02	32.0% (*R*)	

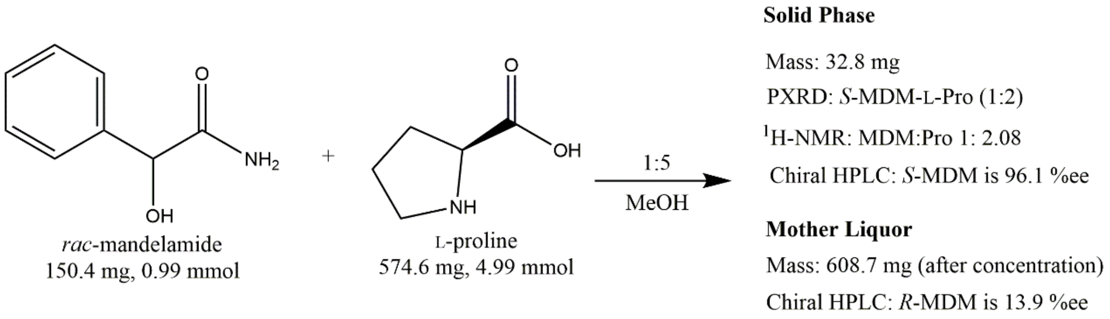

Figure S27. Proof of concept of resolution through cocrystallization.

A preparative batch using 1:5 ratio of (±)-MDM and L-Pro was undertaken and the solid and mother liquor analyzed by HPLC and NMR, and masses of the solid phase and the concentrated mother liquor were determined. As shown in Figure S27, the solid phase consists almost entirely of *S*-MDM-L-Pro (1:2) with excellent enantiopurity of 96.1 %ee for *S*-MDM.

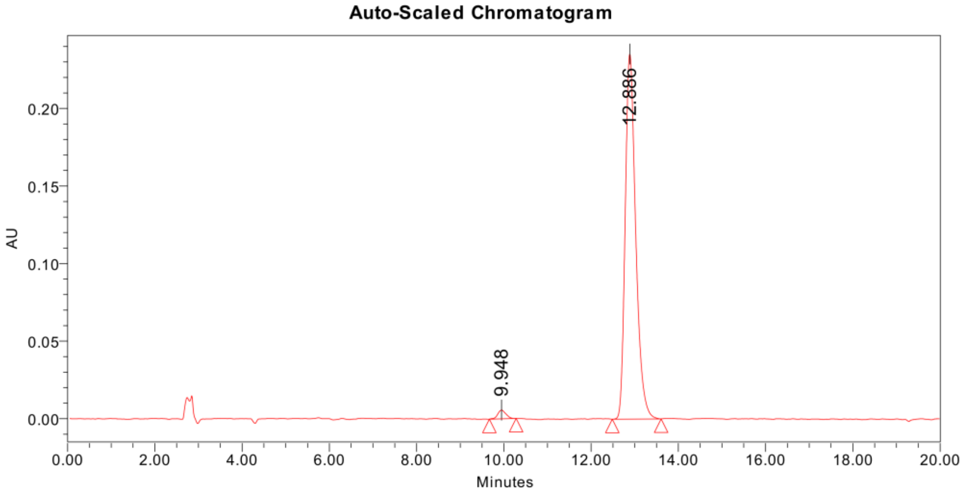

Processed Channel: 2998 PDA 210.0 nm (2998 (210-400)nm)

	Processed Channel	Retention Time (min)	Area	% Area	Height
1	2998 PDA 210.0 nm (2998 (210-400)nm)	9.948	73542	1.83	5653
2	2998 PDA 210.0 nm (2998 (210-400)nm)	12.886	3934596	98.17	235442

Figure S28. Chiral HPLC analysis of the solid *S*-MDM-L-Pro (1:2) recovered from 1:5 experiment (96.3 %ee). The reported result of 96.1 %ee is an average of three HPLC analyses.

Table S11. Summary of reported cocrystals of mandelic acid and chiral coformers.

API	CCF	Crystal structures
Et-DL-mandelate[5] 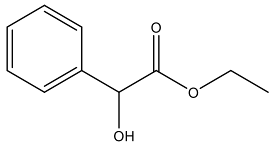	DL-Mandelic acid	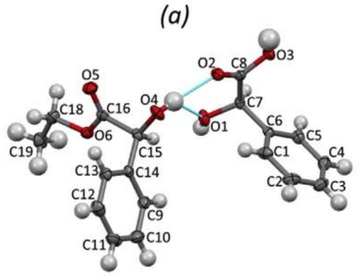
S-Etiracetam 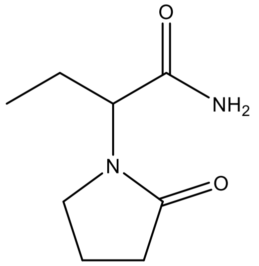	*S*-2-Chloromandelic acid[6]	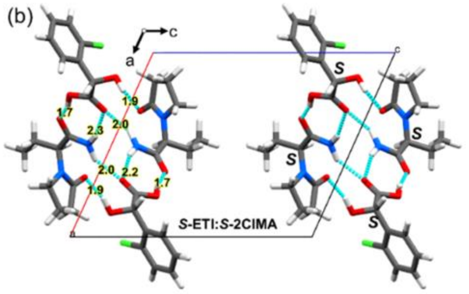
	S-Mandelic acid[7]	No crystal structure
	(±)-4-Bromomandelic acid[8]	
	(±)-2-/3-/4-Chloromandelic acid[8]	
	(±)-4-Fluoromandelic acid[8]	
	(±)-Mandelic acid[8]	
L-Proline[9] 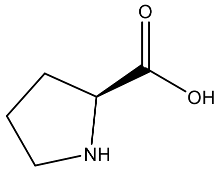	*S*-Mandelic acid (1:1)	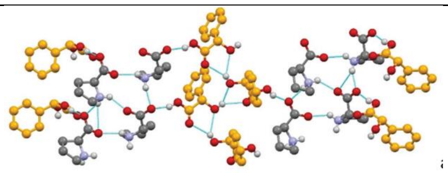
	S-Mandelic acid (2:1)	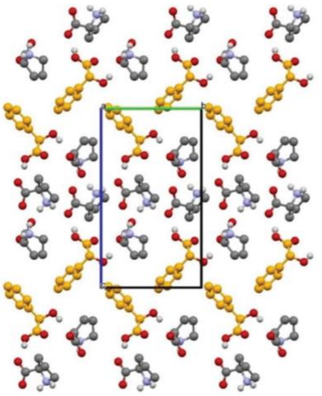

	R-Mandelic acid (1:1)	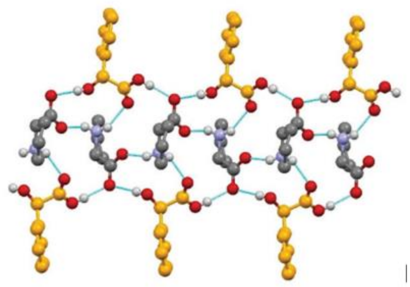
R-Proline amide[10] 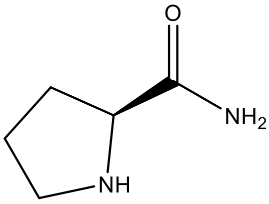	*S*-Mandelic acid	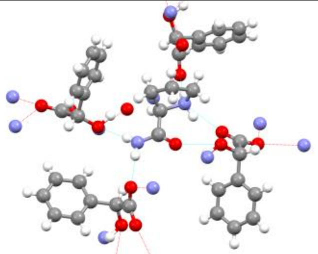
	R-Mandelic acid	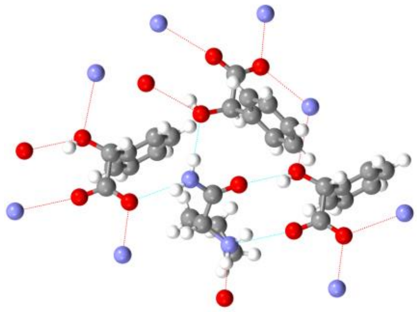
(1*R*,3*S*)-camphoramic acid[11] 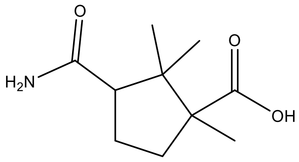	*R*-Mandelic acid	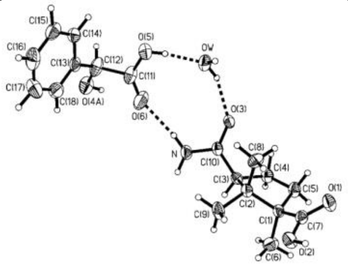
	S-Mandelic acid	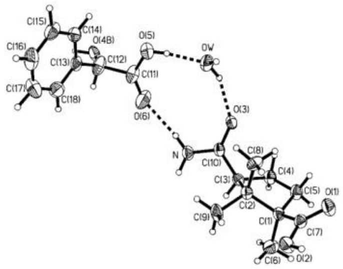
(*R*)-2-tert-butyl-3-methylimidazolidin-4-one[12] 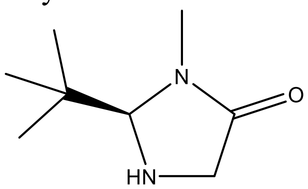	*S*-Mandelic acid	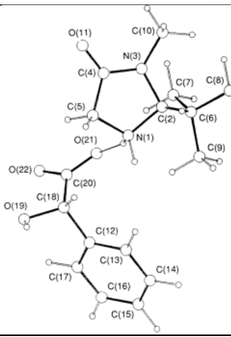

<table>
<tr><td></td><td>R-Mandelic acid</td><td>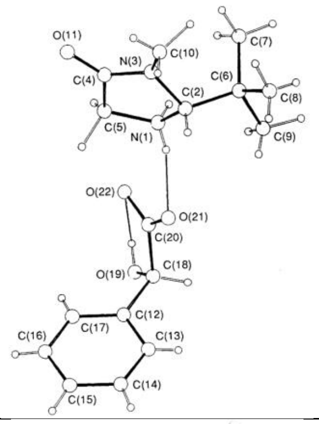
</td></tr>
<tr><td rowspan="2">D-Phenylalanine
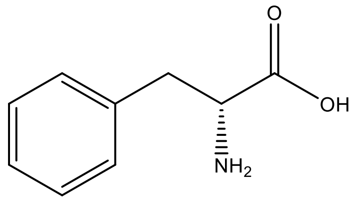
</td><td>R-Mandelic acid[13]</td><td>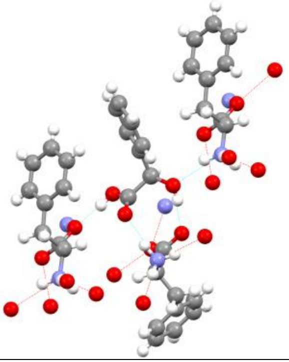</td></tr>
<tr><td>S-Mandelic acid[14]</td><td>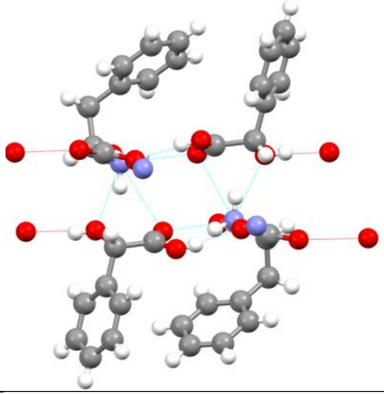</td></tr>
<tr><td>L-Phenylalanine[14]
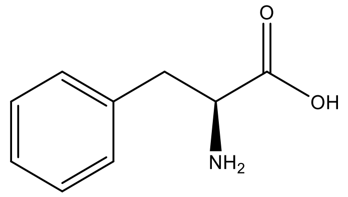
</td><td>S-Mandelic acid</td><td>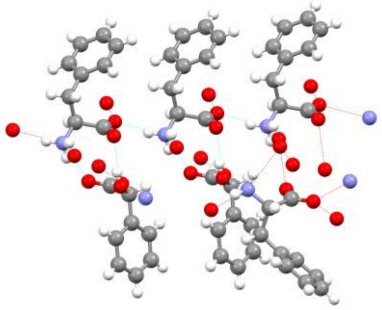</td></tr>
</table>

L-(*R*)-Cysteine[15] 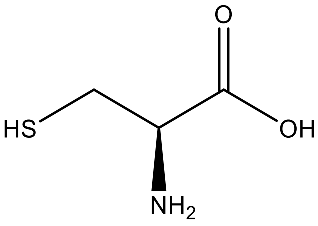	*S*-Mandelic acid	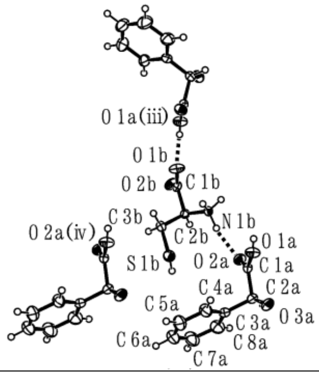
	R-Mandelic acid	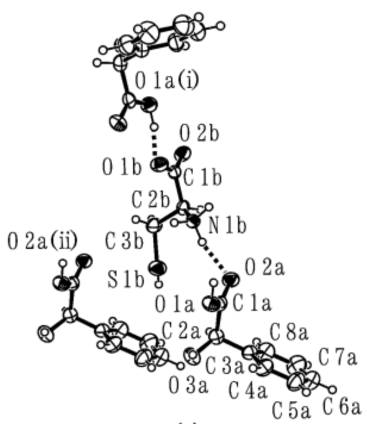
S-Alanine 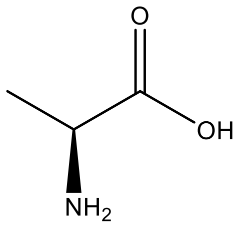	*S*-Mandelic acid[16]	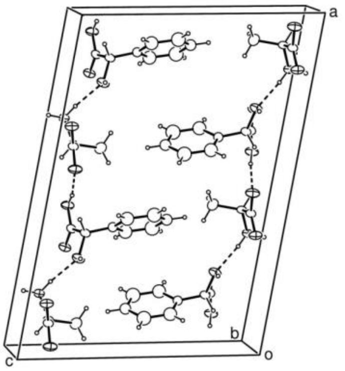
	R-Mandelic acid[17]	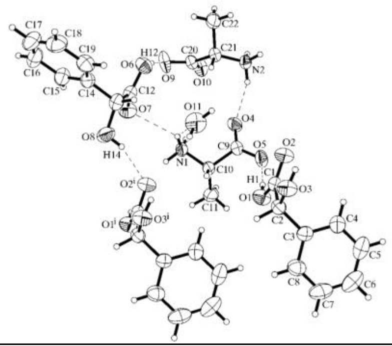

L-(*S*)-Tryptophane[18] 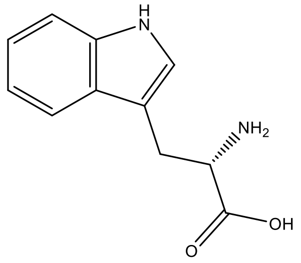	*R*-Mandelic acid	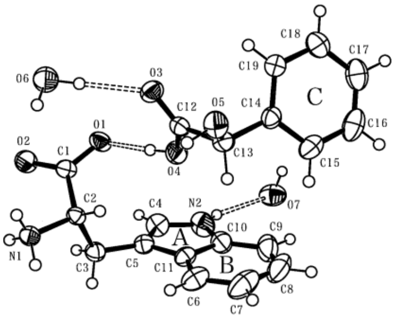
R-Methioninium[19] 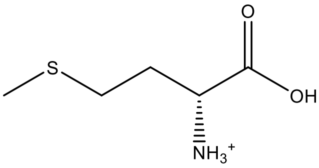	*R*-Mandelic acid	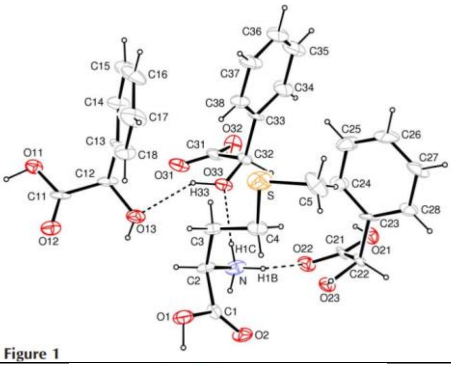 Figure 1
Lamivudine[20] 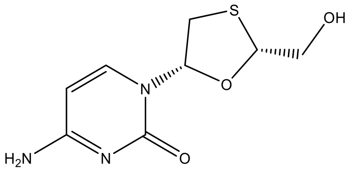	*R*-Mandelic Acid	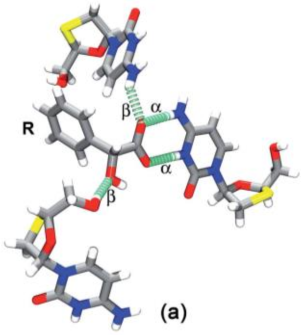
	S-*R*-*S*-Mandelic Acid	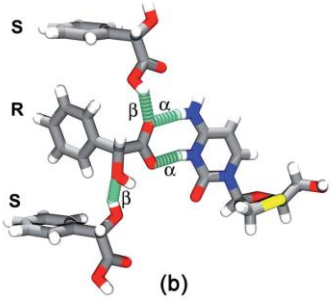
Lvabradine Hydrochloride[21] 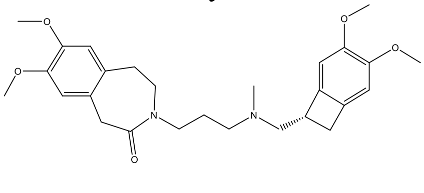	*S*-Mandelic Acid	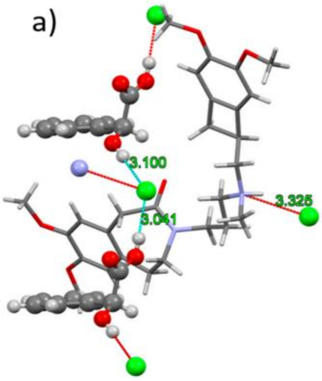

	R-Mandelic Acid	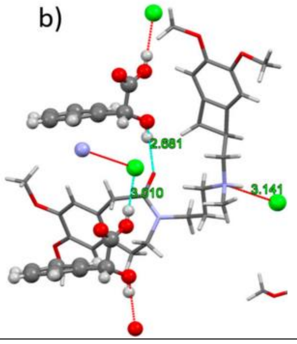
Baclofen[22] 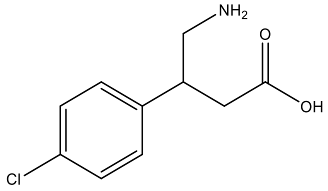	*S*-Mandelic acid	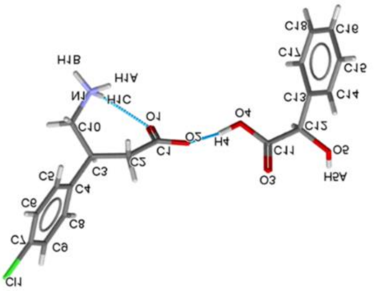
3-(methylamino)-1-(2-thienyl)propan-1-ol[23] 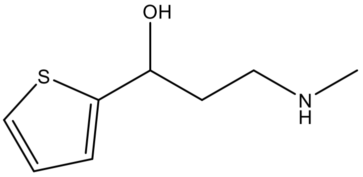	*S*-Mandelic acid	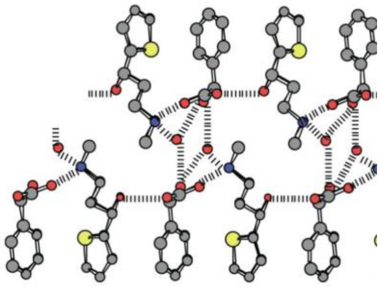
R-2-aminobutanoic acid[24] 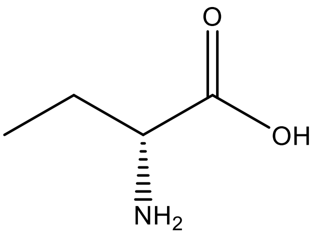	*S*-Mandelic acid	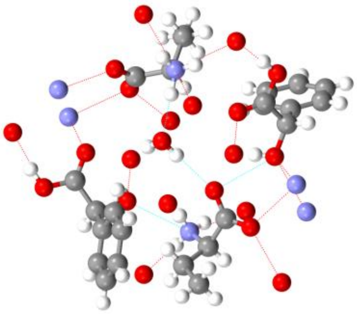
S-1-Phenylethylammonium[25] 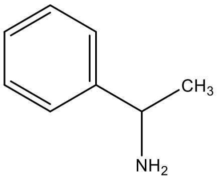	*S*-Mandelic acid	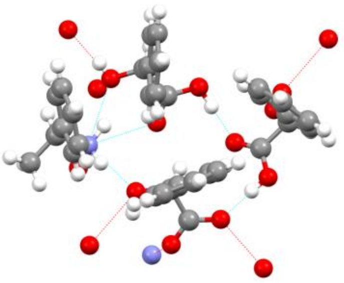

R-1-Phenylethylammonium[26] 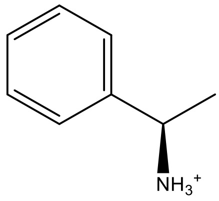	*S*-Mandelic acid	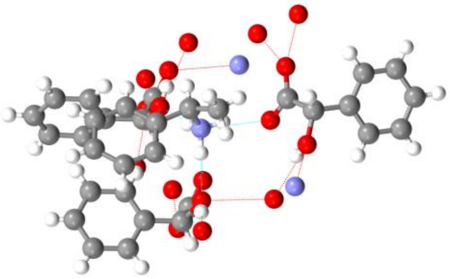
	R-Mandelic acid[27]	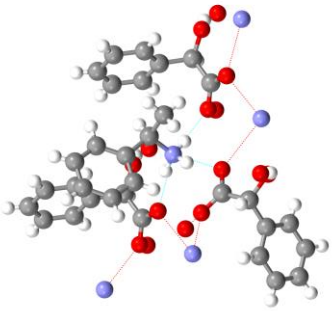
Erythro-2-amino-1,2-diphenylethanol[28] 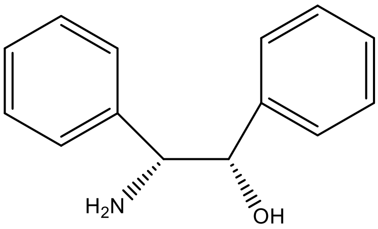	*S*-Mandelic acid	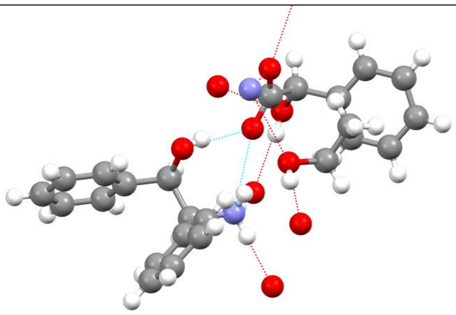
Semotiadil[29] 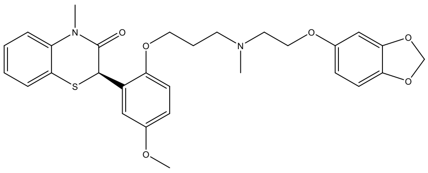	*S*-Mandelic acid	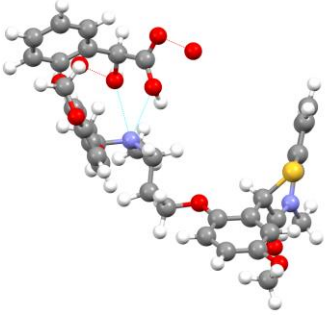
Pregabalin[30] 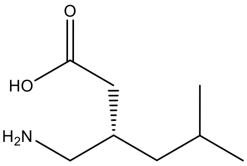	*S*-Mandelic acid	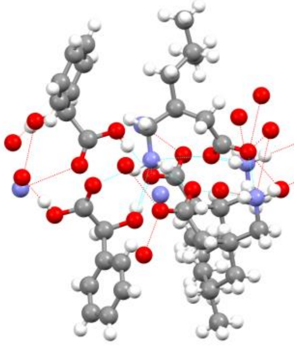
3-(azaniumylmethyl)-5-(ethylsulfanyl)hexanoate[31] 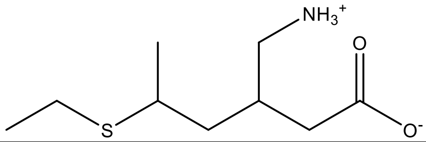	*S*-Mandelic acid	No crystal structure

References:

(1) Lloyd-Jones G.C.; Wall P. D.; Slaughter J.L.; Parker A.J.; Laffan D.P., Enantioselective homoallyl-cyclopropanation of dibenzylideneacetone by modified allylindium halide reagents—rapid access to enantioenriched 1-styryl-norcarene, *Tetrahedron*, **2006**, 62, 11402–11412.

(2) Ebbers E.J.: Ariaans G.J.; Bruggink A.; Zwanenburg B., Controlled racemization and asymmetric transformation of α-substituted carboxylic acids in the melt, *Tetrahedron: Asymmetry*, **1999,** 10, 3701–3718.

(3) Bai J.; Zambron K.; Vogel P., Amides in one pot from carboxylic acids and amines via sulfinylamides, *Organic Letters*, **2014,** 16, 604–607.

(4) Mandelamide|C8H9NO2|Chemspider. https://www.chemspider.com/Chemical-Structure.66240.html (accessed 2023-06-09).

(5) Tumanova, N.; Payen, R.; Springuel, G.; Norberg, B.; Robeyns, K.; Duff, C. L.; Wouters, J.; Leyssens, T., Cocrystallization out of the blue: DL-mandelic acid/ethyl-DL-mandelate cocrystal. *J. Mol. Struct.* **2017,** 1127, 397-402.

(6) Nulek, T.; Klaysri, R.; Cedeno, R.; Nalaoh, P.; Bureekaew, S.; Promarak, V.; Flood, A. E., Separation of Etiracetam Enantiomers Using Enantiospecific Cocrystallization with 2-Chloromandelic Acid. *ACS Omega* **2022,** 7, (23), 19465–19473.

(7) Springuel, G.; Leyssens, T., Innovative Chiral Resolution Using Enantiospecific Co-Crystallization in Solution. *Cryst. Growth Des.* **2012,** 12, (7), 3374–3378.

(8) Wang, J.; Peng, Y., Resolution of Halogenated Mandelic Acids through Enantiospecific Co-Crystallization with Levetiracetam. *Molecules* **2021,** 26, (18), 5536.

(9) Zhou, F.; Collard, L.; Robeyns, K.; Leyssens, T.; Shemchuk, O., L-Proline, a resolution agent able to target both enantiomers of mandelic acid: an exciting case of stoichiometry controlled chiral resolution. *Chem. Commun.* **2022,** 58, 8560-8563.

(10) Bock, D. A.; Lehmann, C. W., Chirality determination from X-ray powder data—diastereomeric co-crystals of mandelic acid and proline amide. *CrystEngComm* **2012,** 14, 1534-1537.

(11) Hu, Z.-Q.; Nie, J.-J.; Xu, D.-J.; Xu, Y.-Z.; Chen, C.-L., The crystal structure of the molecular complex of camphoramic acid with mandelic acid: A partial separation of a racemic mixture of mandelic acid by (1R,3S)-camphoramic acid.

J. Chem. Crystallogr. **2001,** 31, 109–113
(12) Acs, M.; Novotny-Bregger, E.; Simon, K.; Argay, G., Structural aspects of optical resolutions. Optical resolution of (R,S)-mandelic acid. DSC and X-ray studies of the diastereoisomeric salts. *J. Chem. Soc., Perkin Trans. 2* **1992**, 2011-2017.
(13) Hu, Z.-Q.; Xu, D.-J.; Xu, Y.-Z.; Wu, J.-Y.; M.Y.Chiang, *Chin. J. Struct. Chem.,* **2004,** 23, 38.
(14) Okamura, K. A.; Hiramatsu, H.; Nishimura, N.; Sato, T.; Hashimoto, K., Crystal Structures of Diastereomeric 1:1 Complexes of (R)-and (S)-Phenylalanine (S)-Mandelic Acid. *Anal. Sci.* **1997,** 13, 315.
(15) Fujii, I.; Baba, H.; Takahashi, Y., Crystal Structures of L-(R)-Cysteine-Mandelic Acid Diastereomers. *Anal. Sci.: X-Ray Struct. Anal. Online* **2005,** 21, x175-x176.
(16) Hu, Z.-Q.; Xu, D.-J.; Xu, Y.-Z., (S)-Alanine–(S)-mandelic acid (1/1). *Acta Crystallogr., Sect. E: Struct. Rep. Online* **2004,** 60, o269-o271.
(17) Hu, Z.-Q.; Xu, D.-J.; Xu, Y.-Z.; Wu, J.-Y.; Chiang, M. Y., (R)-Mandelic acid (S)-alanine hemihydrate. *Acta Crystallogr. C Struct. Chem.* **2002,** 58, o612-o614.
(18) Fujii, I., Crystal Structure of L-(S)-Tryptophane D-(R)-mandelate 1.5H_2O. *Anal. Sci.: X-Ray Struct. Anal. Online* **2009,** 25, 35-36.
(19) Su, J.-R.; Xu, D.-J., (R)-Methioninium–(R)-mandelate–(R)-mandelic acid (1/1/2). *Acta Crystallogr. E: Crystallogr. Commun.* **2005,** 61, o1933-o1935.
(20) Silva, C. C.; Martins, F. T., The enantiopreference in the solid state probed in lamivudine crystal forms with mandelic acid. *RSC Adv.* **2015,** 5, 20486-20490.
(21) Sládková, V.; Dammer, O.; Sedmak, G.; Skořepová, E.; Kratochvíl, B., Ivabradine Hydrochloride (S)-Mandelic Acid Co-Crystal: In Situ Preparation during Formulation. *Crystals* **2017,** 7, (1), 13.
(22) Songsermsawad, S.; Nalaoh, P.; Promarak, V.; Flood, A. E., Chiral Resolution of RS-Baclofen via a Novel Chiral Cocrystal of R-Baclofen and L-Mandelic Acid. *Cryst. Growth Des.* **2022,** 22, (4), 2441–2451.
(23) Sakai, K.; Sakurai, R.; Yuzawa, A.; Kobayashi, Y.; Saigo, K., Resolution of 3-(methylamino)-1-(2-thienyl)propan-1-ol, a new key intermediate for duloxetine, with (S)-mandelic acid. *Tetrahedron: Asymmetry* **2003,** 14, 1631.
(24) Teng, M.-Y.; Sun, J.; Ma, C.-A.; Song, Q.-B., (S)-2-Hydroxy-2-phenyl-acetic acid–(R)-2-amino-butanoic acid–water (1/1/0.5). *Acta Crystallogr., Sect. E:*

Struct. Rep. Online **2006,** 62, o2454.

(25) Diego, H. L., (S)-1-Phenylethylammonium (S)-Mandelate–Mandelic Acid (1/2), $C_8H_{12}N^+.C_8H_7O3^-.2C_8H_8O_3$. *Acta Crystallogr., Sect. C: Cryst. Struct. Commun.* **1995,** 51, 253-256.

(26) Larsen, S.; Diego, H. L., Isolation of different enantiomers caused by variation in the stoichiometric ratio of racemate and resolving agent. The crystal structure of (R)-1-phenylethylammonium (S)-mandelate·dimandelic acid. *J. Chem. Soc., Perkin Trans. 2,* **1993**, 469-473.

(27) Karamertzanis, P. G.; Anandamanoharan, P. R.; Fernandes, P.; Cains, P. W.; Vickers, M.; Tocher, D. A.; Florence, A. J.; Price, S. L., Toward the Computational Design of Diastereomeric Resolving Agents: An Experimental and Computational Study of 1-Phenylethylammonium-2-phenylacetate Derivatives. *J. Phys. Chem. B* **2007,** 111, (19), 5326–5336.

(28) Shitara, H.; Shintani, T.; Kodama, K.; Hirose, T., Solvent-Induced Reversed Stereoselectivity in Reciprocal Resolutions of Mandelic Acid and erythro-2-amino-1,2-diphenylethanol. *J. Org. Chem.* **2013,** 78, (18), 9309–9316.

(29) Ota, A.; Kawashima, Y.; Ohishi, H.; Ishida, T., Conformational Studies of Semotiadil (SD-3211), a Novel Ca^{2+} Antagonist. *Chem. Pharm. Bull.* **1993,** 41, 1681-1685.

(30) Samas, B.; Wang, W.; Godrej, D. B., 1:1 Cocrystal of (S)-3-(ammonio-meth-yl)-5-methyl-hexa-noate and (S)-mandelic acid. *Acta Crystallogr. E: Crystallogr. Commun.* **2007,** 63, o3938.

(31) Shimada, K.; Ohata, Y.; Kobayashi, J.; Onishi, Y.; Kawamura, A.; Domon, Y.; Arakawa, N.; Inoue, T.; Kitano, Y.; Matsuda, F.; Abe, Y.; Deguchi, T., Alkylsulfanyl analogs as potent $\alpha_2\delta$ ligands. *Bioorg. Med. Chem. Lett.* **2018,** 28, 2000-2002.
